# Crystallization Behavior of Plant-Based Fat Blends Formulated as an Alternative for Anhydrous Milk Fat in Milk Chocolate

**DOI:** 10.1021/acs.cgd.5c00227

**Published:** 2025-04-04

**Authors:** Cecilia Fiore, Tom Rutherford, Francesca Giuffrida, Cynthia Marmet, Elena Simone

**Affiliations:** † Department of Applied Science and Technology (DISAT), Politecnico di Torino, Torino 10129, Italy; ‡ Nestlé Product Technology Centre Confectionery, Haxby Road, York YO31 8TA, U.K.; § Nestlé Research, Vers-chez-les-Blanc, Lausanne 26 1000, Switzerland; ∥ School of Food Science and Nutrition, Food Colloids and Bioprocessing Group, University of Leeds, Leeds LS29JT, U.K.

## Abstract

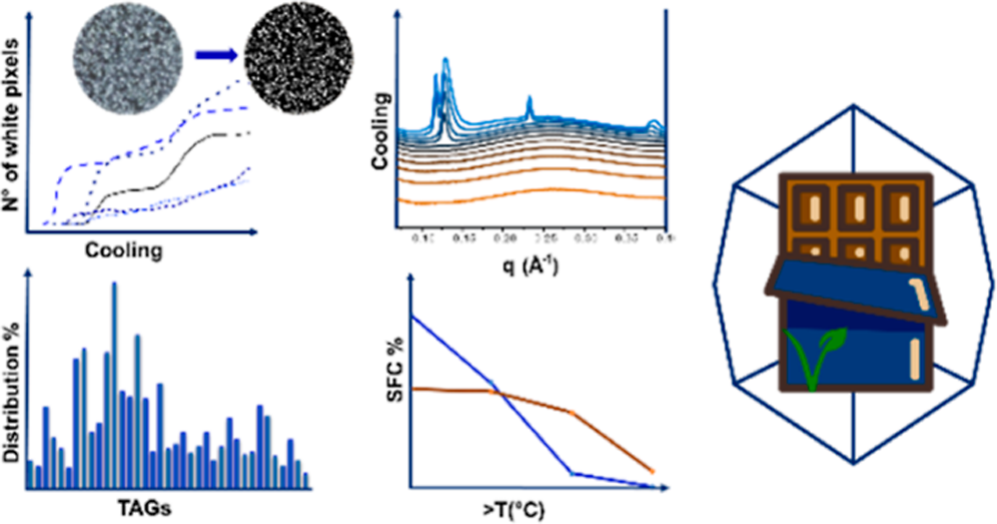

Due to the increasing global demand for chocolate products and changes in consumer preferences, chocolate manufacturers have recently started to explore novel solutions to reformulate chocolate. Milk fat alternatives (MFA) are blends of triglycerides from different plant-based sources that resemble anhydrous milk fat in physical properties, particularly thermal behavior and solid fat content. However, in order to use MFA as potential ingredients for vegan milk chocolate formulations, it is necessary to understand their crystallization behavior, particularly in light of their chemical composition. Here, we applied synchrotron X-ray scattering, polarized light microscopy, and differential scanning calorimetry to investigate the crystallization behavior of four selected commercial MFAs (MF1, 2, 3, and 4), on their own and mixed with cocoa butter (CB). Chemical characterization revealed significant differences among samples and with both anhydrous milk fat (MF) and CB. POP-rich MF1 presented a specific polymorphic and thermal behavior, with the unstable β′ form persisting for longer times than all other samples. Sample MF2 exhibited a polymorphic behavior more similar to CB in terms of number, type, and melting behavior due to the compositional similarities (e.g., prevalence of both SOS and POP). SOS-rich MF3 was characterized by metastable forms γ and β′(3L), whereas MF4 at ambient conditions showed only β(2L) forms due to its specific composition. Mixtures of CB and all MFAs behaved similarly to CB and MF mixtures, with good miscibility at ambient temperature and a lower melting point. Despite significant differences in chemical composition, MF4 presented similar solid fat content compared to MF; this is due to the high amount of relatively long chain, unsaturated fatty acids and the broad distribution of different TAGs, which all lower the melting point of this sample.

## Abstract 2

This work investigates the crystallization behavior (polymorphic landscape and thermal properties) of different plant-based mixtures of triacylglycerides that can be used as milk fat replacers in chocolate formulations. X-ray scattering, differential scanning calorimetry, and polarized light microscopy were used as characterization techniques.

## Introduction

Chocolate is a complex food product that is widely consumed throughout the world. It can be described as a fat-continuous crystalline matrix, where triacylglycerides (TAGs) are the key constituents, within which some nonfat solid particles, generally sugar and cocoa powder, are dispersed.^1^ This crystalline fat phase, which defines many of the quality attributes of the final product, is mainly composed of cocoa butter (CB), a natural fat obtained from the seeds of the cocoa tree (*Theobroma cacao*).^1,2^ In dark or plain chocolate, CB is usually the only fat present, whereas anhydrous milk fat (MF) can be added to obtain milk chocolate products with different tastes and textures.^3−7^

A good quality chocolate must have a shiny surface, a good “snap”, good stability on the shelf (no bloom), good balance between solid and liquid densities (for effective release from the mold), and a melting point that makes it pleasant in the mouth, without a feeling of waxiness.^8−11^ All these characteristics are influenced by the crystal structure of the fat component of the chocolate.^12,13^ CB is a plant-based fat that is a complex mixture of TAGs, and its crystallization behavior can vary accordingly to its variable composition.^14^ In fact, depending on the origin of the CB sample, the fatty acid and TAGs composition may slightly change.^15^ However, usually CB is largely composed of palmitic acid (16:0), stearic acid (18:0), and oleic acid (18:1) in the second position and, in lower quantity, of linoleic acid (18:2) and arachidic acid (20:0). This fatty acid composition generally results in three main triglycerides: 1(3)-palmitoyl-2-oleoyl-3(1)-stearoyl glycerol (POS), 1,3-dipalmitoyl-2-oleoyl glycerol (POP), and 1,3-distearoyl-2-oleoyl glycerol (SOS).^16,17^ CB can form six different polymorphs, namely, form I to form VI from the least stable to the most stable. While form I and II are liquid crystalline, the others are semisolid with increasing melting points. CB polymorphs are characterized by different stacking modes (2L or 3L), different unit cells (hexagonal α, orthorhombic β′, or monoclinic β), and hence different physical properties.^1,9,18^ Only form V has the desired organoleptic properties for chocolate; hence, a good control over the crystallization process is essential for the production of a good quality chocolate.^11^

Milk fat is one of the most complex fats in terms of composition, which varies widely depending on the feeding type and genetic differences of the species from which it is obtained.^19^ It contains a variety of different fatty acids with a wide range of carbon chain lengths. One of the peculiar features of milk fat is the presence of a non-negligible amount of short-chain fatty acids (4:0 and 6:0). These are usually located on the sn-3 carbon of the TAG molecule. Both the amount and location of these shorter-chain fatty acids in the TAG molecules affect milk fat properties, such as melting point and flavor.^28^ The most prevalent TAGs found in milk fat^26^ are reported in Table 1, with their respective concentration (mol %). Anhydrous milk fat has its own specific crystallization behavior;^22^ however, when mixed with CB to prepare a milk chocolate fat blend (e.g., up to 50% milk fat), the same polymorphs of CB are observed, albeit with lower melting points due to formation of eutectic mixtures.^21^

**Table 1:** Concentration of the Most Prevalent TAGs in a Milk Fat Sample^26^^a^

TAG	concentration (mol %)
BuPO	4.2
BuPP	3.2
BuMP	3.1
MPO	2.8
POO	2.5
BuPS	2.5
PPO	2.3
PSO	2.2
CaPO	2.0
BuMO	1.8

a Bu stands for butyric acid, P for palmitic, M for myristic, S for stearic, O for oleic, and Ca for caproic acid.

In the last few years, the cost of chocolate production has increased significantly, due to the increase in the cost of cocoa, as well as the steadily increasing global demand of chocolate products.^2^ At the same time, consumers’ taste and need have changed, with trends like veganism becoming more and more popular. Hence, significant effort has been made to find ways to replace CB and/or MF in confectionary products to decrease production costs and meet consumers’ demands, while maintaining the same organoleptic properties and ensuring process and product sustainability.^2−5^ One of the possible strategies is to partly or fully replace these two fats with different plant-based fat mixtures (cocoa butter and milk fat alternatives, CBAs, MFAs) that resemble CB and MF in their thermal properties (e.g., solid fat content) and texture.^2,25−27^ However, despite their similar properties, cocoa butter and milk fat alternatives have a significantly different TAG composition compared to CB and MF, which can affect their crystallization behavior (e.g., number and type of polymorphs in the final product) and, hence, the properties of the final chocolate product.^25,27−29^ Therefore, understanding the relationship between TAG composition and crystallization behavior of CB and MF alternatives is essential for an effective selection of the most suitable chocolate ingredients and the design of new chocolate recipes.^23,24^

In this work, the crystallization behavior (polymorphic landscape and thermal properties) of four plant-based TAG mixtures commercialized as milk fat alternatives (named MF1, MF2, MF3, MF4, and MF*n*) was studied and compared to that of commercial anhydrous milk fat. Pure MF*n* and its mixtures with CB in ratios typical of milk chocolate were also characterized.

A combination of synchrotron X-ray scattering, differential scanning calorimetry (DSC), and polarized light microscopy were used to analyze the crystallization behavior of the MF*n* and their blends with CB. Samples were characterized also in terms of their TAGs and fatty acid composition via chromatographic techniques. This information was found to be essential to correctly interpret the structural information provided by X-ray scattering and correlate the observed crystallization behavior with the TAGs composition of each analyzed mixtures. This is crucial not only for creating innovative chocolate recipes incorporating CBEs and MF substitutes but also for understanding the crystallization patterns in other similar blends of TAGs, potentially usable in food, cosmetic, or pharmaceutical contexts.

## Materials and Methods

### Materials

Samples of cocoa butter (CB), anhydrous milk fat (MF), and milk fat alternatives (MF*n*; *n* = 1, 2, 3, and 4) were provided by the Nestlè Product Technology Centre Confectionery in York (UK). CB is a cocoa butter sample of Ghanian origin, and MF is an anhydrous milk fat sample derived from cow milk (Cargill, UK). MF*n* are commercial TAGs plant-based mixtures; MF1 is based on fractionated palm and shea oil, while MF3 is based on exotic oils (e.g., argan, marula, and chia). MF2 is a fractionated, non-hydrogenated, refined vegetable fat of nonlauric origin. MF4 is a blend of fractionated and non-hydrogenated vegetable oils. All samples present different solid fat contents (provided by the suppliers), as shown in Table 2, which are compatible with cocoa butter: MF*n* (*n* = 1, 2, 3, and 4) samples were analyzed and studied in the meaning of their possible use as MF alternatives in a novel formulation of a vegan, lactose-free and more sustainable chocolate product. Their blends in a 20% proportion of MF*n* in CB were also analyzed in comparison to a blend of 20% of MF in CB.

**Table 2:** Solid Fat Contents for All MF*n* (*n* = 1, 2, 3, and 4) at Different Temperatures^a^

temperature	MF1	MF2	MF3	MF4
20 °C	65	42–51	37	25
25 °C	39	35–45	36	17
30 °C	5	23–30	28	7
35 °C	0	0–6	6	3

a Information provided by the suppliers and reported on the datasheets of the products.

### Preparation of Cocoa Butter, Milk Fat, and Milk Fat Alternatives Mixtures

5 g of each sample was melted in a preheated oven at 70 °C and kept for 1h to ensure complete melting. Mixtures with 20% mass of MF and MF*n* in CB (1 g of MF/MF*n* in 4 g of CB) were prepared by weighing on a scale the required mass of each melted sample using a pipette and placing them in 10 mL glass vials. The blends were then heated up again at 70 °C and kept for another hour in the oven for a better mixing of the melted constituents.

### Chemical Characterization

CB, MF, and MF*n* were characterized in terms of fatty acid (FA) and triacylglyceride composition by gas chromatography. An Accela 1250 liquid chromatograph (Thermo Fisher Scientific, Bremen, Germany) equipped with an Agilent Poroshell 120 EC-C18 (2.7 μm particle size, 2.1 × 250 mm) was used for separation of analytes, while an LTQ-Orbitrap XL hybrid mass spectrometer (Thermo Fisher Scientific, Bremen, Germany) was used for the identification of the TAGs and FAs that characterized each sample. The detailed experimental procedure is described elsewhere.^30^ The TAG composition of milk fat was evaluated according to the Zeb and Murkovich procedure^31^ in a Shimadzu LCMS 2020 (Japan) with a separation column Phenomenex Luna 3u C18(2) 100A LC Column 150 mm × 3.0 mm. The detailed procedure can be found in our previous work.^19^ Measurements were performed in triplicate, and the collected data were processed in the LabSolutions software (version 5.97) to obtain chromatograms and mass spectra. Further chromatograms peak fittings, integration, and plotting were carried out using OriginPro 2019b. Prior to fitting, the solvent contribution was removed.

### Differential Scanning Calorimetry

The differential scanning calorimetry (DSC) curves were obtained using a Mettler Toledo DSC 1 (Mettler Toledo, USA). Approximately 3 mg of each sample was weighed and sealed in an aluminum pan, while an empty aluminum pan was used as a reference. Each sample was heated up to 70 °C with a rate of 10 °C/min, kept at such temperature for 5–10 min, cooled to −10 °C at −5 °C/min, then reheated to 70 °C at 2 °C/min (isothermal for 5–10 min), and finally cooled to −10 °C at −2 °C/min. The heating and cooling rates were selected in order to optimize the thermal signal and better identify the onset and end temperature of melting and recrystallization. DSC traces are reported in Supporting Information, Section 4.

### Polarized Light Microscopy

**Figure 1:**
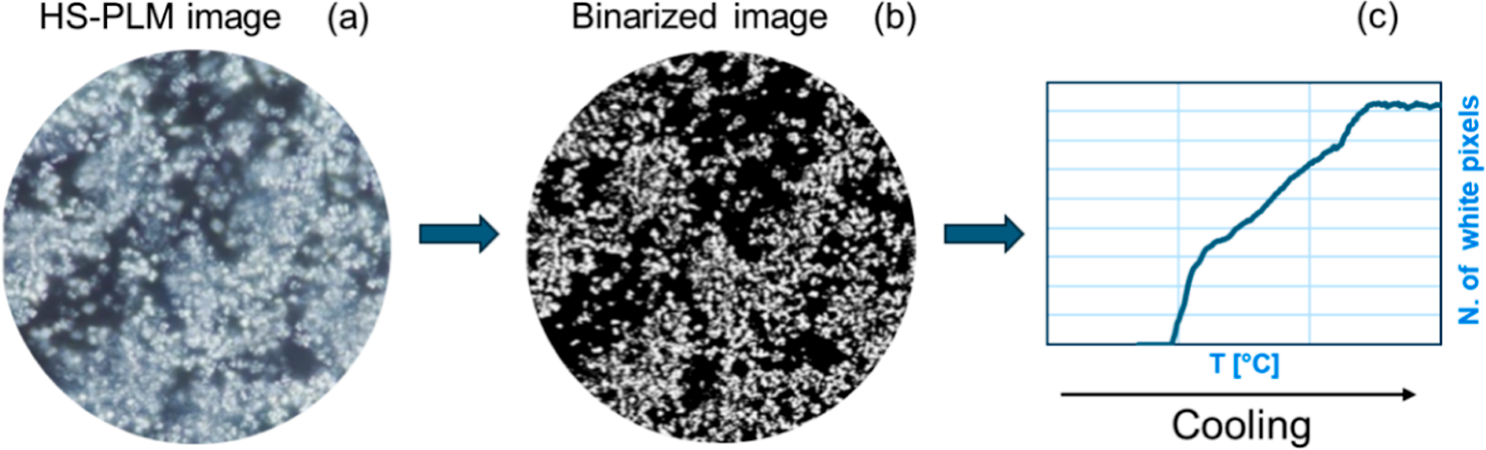
Graphical example of the image processing methodology applied in this work: frames are taken at different temperatures from the video recorded during a crystallization experiment with the HS-PLM (a), then the images are processed and binarized in black and with pixels (associated with crystals) (b), and from the count of pixels in each frame, it is possible to obtain a plot where the number of pixels (corresponding to the solid crystalline material) is expressed as a function of the temperature (c).

Samples were heated in an oven at 50 °C until they were fully molten. Approximately one drop of each sample was transferred on a glass slide using a plastic pipette and then covered with a cover glass. The glass slide was then placed onto a Linkam PE120 hot stage and connected to a water circulation pump (Linkam Scientific Instruments, UK). The hot plate was positioned on a Zeiss Axiolab 5 microscope (Zeiss, Germany) with a polarized light lens. The temperature was then varied with a T96 Peltier LinkPad controller. Each sample was heated up to 70 °C at 10 °C/min, kept at such temperature for 5–10 min, then cooled down to 5 °C at −2 °C/min, heated again to 70 °C at 0.5°/min, kept under isothermal conditions for 5–10 min, and finally cooled to 5 °C at −0.5 °C/min. Videos of the crystallization and melting of the samples were recorded with an iPhone 13 camera setup (Apple, USA), using the 40× magnification lens of the microscope. Figure 1 shows a graphical example of the image processing methodology applied. Videos and images were then processed using Matlab R2023a, by extracting a frame every 30 s. For each extracted frame, the background was removed, and then the truecolor image was converted into a grayscale and then into a binary one (the threshold level was selected manually for each sample). The binarized images were composed of just black and white pixels, the latter representing the crystals; hence, the amount of white pixels is related to the amount and size of crystals formed. By plotting the number of white pixels in a specific image as a function of time, it is then possible to follow the crystallization process.

### Powder X-ray Diffraction

Powder X-ray diffraction (PXRD) patterns for CB and MF were recorded with an Empyrean diffractometer (Malvern Panalytical, U.K). Diffraction patterns in the wide-angle region (2θ range of 4–40°) were obtained using Cu K_α_ radiation with a wavelength (λ) of 1.54 Å. The instrument operated with an intensity of 40 mA and a voltage of 40 kV. The solid samples kept at ambient temperature for several weeks (i.e., at equilibrium) were gently grinded in an agate mortar and pestle before placing them onto a sample holder. Gentle grinding prevented undesired heating and melting of the samples before the measurement. The samples with a lower solid fat content at room temperature were directly pressed into the sample holder without grinding. The wide angles were converted to *d*-spacing values using Bragg’s Law , resulting in a range of 2.25–22 Å.

### Synchrotron Small and Wide-Angle X-ray Scattering

SAXS and WAXS measurements were performed at the SAXS/WAXS beamline at the Elettra Sincrotrone facility in Trieste (Italy) and at the I22 beamline of the Diamond Light Source (Didcot, UK). The energies of the beam used were 10 and 18 keV, respectively. At Elettra, the SAXS patterns, in the range of q of 0.04–0.72 Å^–1^, were recorded by a Pilatus3 1 M detector (Dectris Ltd., Switzerland). At Diamond SAXS, 2D diffraction patterns were recorded on a Pilatus 2 M detector (Dectris Ltd., Switzerland) in the range of *q* of 0.0015–0.18 Å^–1^. For WAXS, a Pilatus P3-2M-DLS-L (silicon hybrid pixel detector, Dectris Ltd., Switzerland) was used at Diamond, and a Pilatus 100K (Dectris Ltd., Switzerland) was used at Elettra. For both synchrotron setups, quartz capillaries were filled with the melted samples and placed at room temperature for 20 days before the experiments. The experiment temperature was varied using a Peltier element, and temperature profiles used are reported in Table 3. The exposure times were 1 s for beamline I22 and 20 s for Elettra SAXS beamline. The data obtained from SAXS and WAXS measurements were analyzed using Origin2018 (OriginLab). In order to resolve overlapping peaks, small portions of data were analyzed individually, performing baseline correction if necessary and applying fitting routines with Gaussian or Voigt functions (selected based on whichever type of function fitted the data better). Thus, the *q* values of the peaks at different diffraction orders were obtained and used to determine the number and *d*-spacings of the different polymorphs present in the sample. The *d*-spacing is related to the *q* values through the following equation
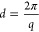
1To distinguish the lamellar phases present in each sample from the SAXS/WAXS data, the values of *q* of every peak were plotted as a function of their diffraction order (or Miller index h value). In these type of plot, *q* values related to a specific lamellar phase lie on a straight line (originating from 0) with a slope equal to the characteristic *d*-spacing of this crystalline phase. If more than one lamellar phase is present, different straight lines can be identified with the *q*-values estimated. Samples in capillaries were heated up from 20 to 70 °C at a rate of 0.5 °C/min and kept at such a temperature for 5–10 min. A first cooling ramp at −0.5 °C/min was set, and samples were cooled to 5 °C. After 30 min of isothermal hold, samples were heated up to 70 °C at 5 °C/min and then cooled again to 5 °C at −5 °C/min (after 5–10 min of holding at high temperature).

**Table 3:** Temperature Profiles Performed during SAXS/WAXS Measurements

*T* (°C) profile	MF*n*/CB/20% blends	MF
20 °C → 50 °C	2 °C/min	2 °C/min
50 °C → 70 °C	10 °C/min	10 °C/min
holding 70 °C	5 min	5 min
70 °C → 45 °C	–10 °C/min	–10 °C/min
45 °C → 5 °C	–0.5 °C/min	–0.5 °C/min
holding 5 °C	20 min	20 min
5 °C → 50 °C	0.5 °C/min	0.5 °C/min
50 °C → 5 °C	–5 °C/min	
holding 5 °C	20 min	

## Results and Discussion

### Chemical Composition of MF, CB, and MF Alternatives

**Figure 2:**
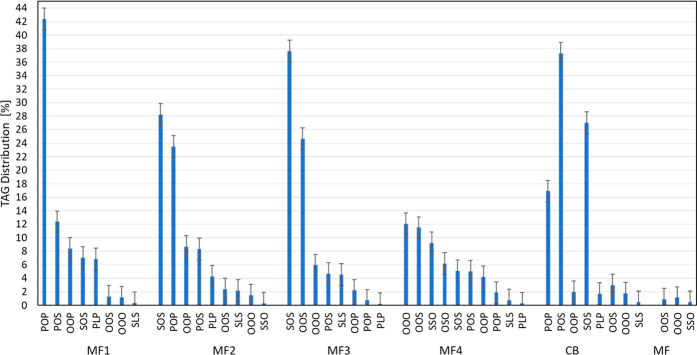
Major TAGs found in compositional characterization of the samples considered in this work.

The MF sample has a complex composition, with a wide variety of fatty acids present, which leads to a complex TAG profile.^26^ This is very different from the general composition of CB, which is usually mainly composed of POS, POP, and SOS triglycerides. In the MF sample used in this work, the TAG in the highest concentration is BuPO (7.2%), followed by BuMP (5.1%), BuPS (4.9%), and BuMO (4.4%). The complete TAG composition of MF is reported in Supporting Information, Section 1, Table 1. The simplified TAG composition of CB, MF, and the MF*n* is instead shown in Tables 4 and 5 and Figure 2.

**Table 4:** TAG Composition of MF*n* and CB Samples Used in This Work^a^

TAG composition (%)	MF1	MF2	MF3	MF4	CB^34^	MF^26^
POP	42.4	23.5	<1	1.9	16.9	3.4
POS	12.4	8.3	4.7	5	37.3	2.3
SOS	7	28.2	37.7	5.1	27	0.6
trisaturated	2.7	2.8	3.2	3.9	<1	7.3
triunsaturated	1.7	2.2	7.6	18.8	1.8	1.7
diunsaturated	13.5	15.4	33.1	31.1	10	13.3

a P stands for palmitic, S for stearic, and O for oleic acid.

**Table 5:** Fatty Acid Composition of MF*n*, CB, and MF Analyzed Samples

fatty acids (FA)	MF1	MF2	MF3	MF4	CB	MF
palmitic (%)	47.6	30	2.8	13.7	25.2	24.4
oleic (%)	31.5	31.9	41.6	46.1	31.4	19.5
stearic (%)	11	26.1	37.4	26.7	34.9	4.9
saturated (%)	58	56	45	38	61	69
unsaturated (%)	37	39	50	57	34	20
average FA chain length (number of C)	17.2	17.4	17.9	17.5	17.5	15

Referring to Table 4, MF1 has the higher amount of POP, followed by MF2 and CB, while MF3 and MF4 present a very low quantity of POP (<1 and 1.9 respectively), similar to MF. MF3 possesses a larger quantity of SOS, and MF2 comes right after, still maintaining a more equilibrated proportion of POP and SOS. In MF3, the high amount of OOS and OOO characterizes the unique profile of this TAG mixture. MF4 has a higher variety in terms of TAGs compared to the other samples; in fact, the respective amount of POP, POS, and SOS is much lower (<6%) and their sum does not exceed the 12% of the total. In MF4, OOO and OOS are the major components, followed by SSO. All the MF*n* samples have a higher percentage of diunsaturated TAGs compared to CB and MF, and among them, MF3 has the largest amount, with 33.1% of the total mixture. MF4 follows with 31.1% of diunsaturated TAGs, and it also has the highest quantity of triunsaturated TAGs (18.8%). In comparison to CB, all four MF*n* also possess a higher percentage of trisaturated TAGs, with the maximum of MF4 (3.9% of its total composition) compared to CB that has less than 1%. Considering our MF sample, which has 7.3% of trisaturated TAGs, all MF*n* and CB have a smaller amount. The relative fatty acids (FA) composition is also crucial. Analyzing the mixture in terms of distribution of saturated and unsaturated FA together with the average chain length allows to get a better understanding of the melting profile and crystallization behavior of the samples. In fact, the melting point generally increases with chain length and decreases with unsaturation. TAGs with long and/or saturated chains have a high melting point, whereas those with polyunsaturated and/or shorter or asymmetric chains have a lower melting point.^32^ The FA composition is summarized in Table 5, which also reports the average chain length for each MF*n*, CB, and MF. It is worth noticing the high amount of saturated FA in MF, which results also in a high percentage of trisaturated TAGs, and the significantly shorter average FA chain of MF compared to the rest of the samples. In fact, the presence of short FA (down to 4C atoms) is responsible for the relatively low melting point and SFC of MF, despite the abundance of saturated FA.^20,32^

### Polymorph Identification with PXRD and SAXS/WAXS Measurements

The polymorphic forms present in the MF*n* solid samples at room temperature (20 °C) were investigated with both small- and wide-angle X-ray scattering/diffraction. Dynamic crystallization experiments were also performed using synchrotron SAXS/WAXS.^30^

**Figure 3:**
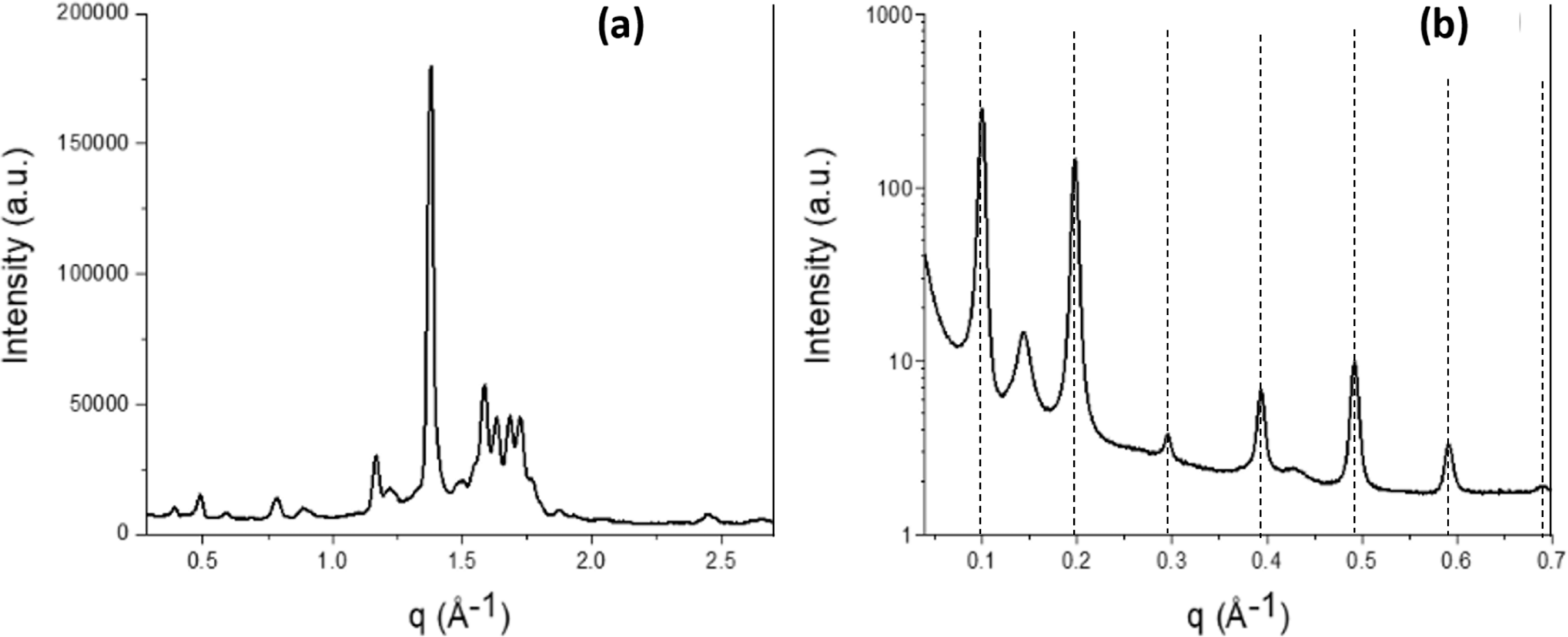
CB PXRD diffractogram at 20 °C (a); CB SAXS diffractogram at 20 °C (b) after at least 1 week of equilibration. Dotted lines indicate the SAXS peaks of the β(*V*) polymorph; the other peaks clearly visible in the SAXS pattern belong to β(2L) forms with a high melting point.

The PXRD diffractogram of CB, shown in Figure 3a, was indicative of the presence of the β(V) polymorph.^33^ However, the SAXS/WAXS data (Figure 3b), (20 °C) showed the presence of two immiscible β polymorphs, in accordance with what was observed by Simone et al.:^34^ the β(*V*) (*d* = 64 Å) and β-2L (*d* = 44 Å) with a higher melting point. SAXS/WAXS patterns of CB collected during cooling, holding, and heating profiles are discussed elsewhere^34^ and reported again in Supporting Information, Section 2.1, Figure 1a–d.

**Figure 4:**
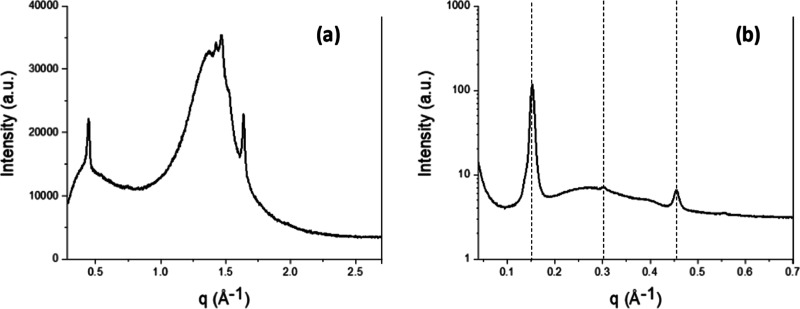
MF PXRD diffractogram at 20 °C (a); MF SAXS diffractogram at 20 °C (b) after at least 1 week of equilibration. Dotted lines indicate the SAXS peaks of the β′(2L) polymorph, and the remaining peaks at *q* values of 0.15, 0.41, and 0.55 Å^–1^ belong the α(2L) form.

The PXRD diffractogram of MF at equilibrium conditions did not possess a clear baseline due to the low solid fat content of MF at room temperature. In Figure 4a, the peaks at *q* = 1.427 Å^–1^ (*d* = 4.5 Å) and 1 = 1.636 Å^–1^ were representative of a β′ polymorph, whereas the weak peak at *q* = 1.475 Å^–1^ (*d* = 4.3 Å) suggested the presence of traces of an α form.^35^ These observations agreed with the SAXS/WAXS pattern shown in Figure 4b where two polymorphs are noticeable, associated with a sharp peak at *q* = 0.152 Å^–1^ and a shoulder at lower values of *q*. The higher peak corresponding to a long spacing of 41 Å was consistent with the *d*-spacing value reported in the literature for the β′(2L) polymorph for milk fat.^19,36^ The weaker peak was likely associated with the α(2L) (*d* = 46 Å) polymorph.^37^ The coexistence of these two polymorphs was more evident at higher ranges of *q*, where the Bragg peaks did not overlap (e.g., the third order peaks of the α and β′ forms at *q* = 0.41 Å^–1^ and *q* = 0.454 Å^–1^). The presence of the α polymorph was also suggested by the peak at *q* = 0.55 Å^–1^, representing the fourth order of diffraction.

The crystallization and melting behavior as a function of the TAGs composition is in accordance with what was previously reported by Pratama et al.^19^ All SAXS/WAXS patterns collected during cooling, holding, and heating profiles are reported in Supporting Information, Section 2, Figure 2a–d.

**Figure 5:**
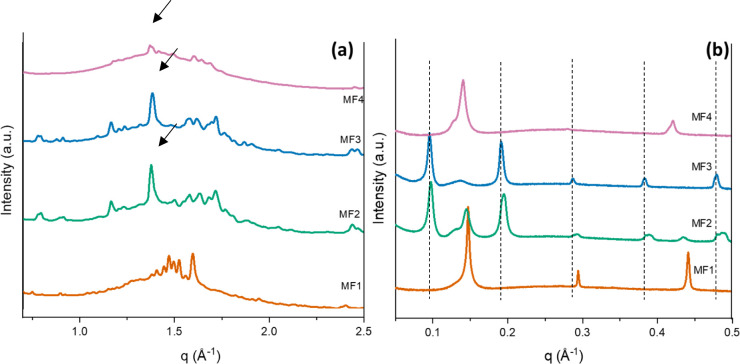
MF*n* WAXS patterns at 20 °C (a); MF*n* SAXS patterns at 20 °C (b) after at least a week of equilibration. Arrows in the WAXS pattern point to the strongest, characteristic β(3L) peaks for MF2 and MF3 and the strongest characteristic β(2L) peak in MF4. The WAXS pattern of MF4 is typical of a β′(2L) polymorph. Dotted lines indicate the indicative positions of the SAXS peaks for a β(3L) polymorph. Other peaks present in the patterns (*q* range between 0.11 and 0.17 Å^–1^) belong to β′(2L) or β(2L) forms depending on the sample.

The polymorphism in MF*n* at room temperature was investigated in the SAXS and WAXS range. From the WAXS results of MF1, in Figure 5a, the peaks at *q* = 1.461 Å^–1^ (*d* = 4.3 Å) and *q* = 1.583 Å^–1^ (*d* = 3.96 Å) are markers of the presence of a β′ polymorph.^35,38^ The MF1 SAXS/WAXS pattern (Figure 5b) indicated that this was indeed a β′(2L) form melting completely at around 37 °C (see Supporting Information, Section 2.3, Figure 3a). Sharp peaks were visible at *q* = 0.149 Å^–1^, *q* = 0.297 Å^–1^, and *q* = 0.445 Å^–1^, representing, respectively, the first-, second-, and third-order reflections of the same polymorph, with a *d*-spacing *d* = 42 Å. The persistence of this polymorph in the POP-rich sample MF1 is in accordance with the previous literature.^34,39^

The SAXS/WAXS pattern of MF2 at 20 °C (Figure 5b) indicated the presence of multiple polymorphs, which were assigned to two β(2L) forms arbitrarily called β1(2L) and β2(2L), with a *d*-spacing of 43 and 47 Å, as shown in Supporting Information, Section 3.4, Figure 4a. The peak with the lowest *q* value (0.09 Å^–1^) and *d*-spacing (68.9 Å) can be assigned to a β(3L) form, whose third-, fourth-, and fifth-order peaks were also visible. The WAXS diffractogram of MF2 at equilibrium conditions (Figure 5a) showed the presence of a sharp peak at 1.46 Å^–1^, and other peaks of medium intensity at 1.37, 1.39, and at 1.54 Å^–1^ associated with this β(3L) form. The presence of other small peaks close to the characteristic β ones was indicative of the coexistence of the other β forms observed in the SAXS range. The β(3L) polymorph melted first at around 36 °C, whereas the two β(2L) forms showed higher melting points of 42.5 and 44 °C (see Supporting Information, Section 2.4, Figure 4a). The polymorphic landscape of MF2 is very similar to pure CB and is related to the high amount of both POP and SOS that characterize both these fat blends, as well as the presence of trisaturated TAGs and molecular compound forming TAG pairs that are characterized by β(2L) structures.

At 20 °C, SOS-rich MF3 showed evidence of the presence of two separate polymorphs: a sharp peak at *q* = 0.098 Å^–1^ and the characteristic WAXS pattern (peak at *q* = 1.365 Å^–1^, *d* = 4.6 Å) are indicative of a β(3L) polymorph with a *d*-spacing of 64 Å (Figure 5). This value is in accordance with the results of a previous study on pure SOS.^40^ Peaks at *q* = 0.194 Å^–1^, *q* = 0.291 Å^–1^, and *q* = 0.388 Å^–1^ represented the second-, third-, and fourth-order reflections for this polymorph. However, the less intense but still evident SAXS peak at *q* = 0.140 Å^–1^ (*d* = 44.9 Å) also suggested the presence of a β(2L) form (this polymorph was attributed to the presence of trisaturated TAGs, due to its high melting point of 45 °C, which is evident in Supporting Information, Section 2.5 Figure 5a).

SAXS/WAXS at 20 °C of the MF4 sample was then analyzed, and MF4 appeared only partially crystalline due to its low SFC at room temperature. As depicted in Figure 5,b the peaks at *q* = 0.140 Å^–1^ and *q* = 0.142 Å^–1^ could be associated with the first reflection of two different lamellar phases. It is important to highlight that these two polymorphs, having different melting points, showed similar *d*-spacing values (*d* = 44.8 and *d* = 44.3 Å), compatible with β(2L) structures, considering the TAGs composition of MF4 and the WAXS diffractogram of MF4 (Figure 5a). These highly stable polymorphs can be attributed to the presence of trisaturated TAGs (SSS and PPP) or 2L molecular compounds formed by specific pairs of TAGs (such as stoichiometric mixtures of SSO or OSO and SOS). This was further confirmed by the high melting point of these polymorphs (47–48 °C).^41−43^

MF2 and MF3 resemble more CB in terms of the polymorphic landscape, as a β(3L) form was detected in both. MF4 molecular composition instead seemed to lead preferentially to 2L structures. The absence of the β form in MF1 suggested that the transformation from the less stable β′ polymorph to the more stable form is slower in this sample compared to the others. The reason for this difference is probably due to the high content of POP. A slower transformation toward the β polymorphs in mixtures rich in POP was previously observed.^31,44,45^

### Crystallization Behavior Studied with SAXS/WAXS under Controlled Temperature Profiles

**Figure 6:**
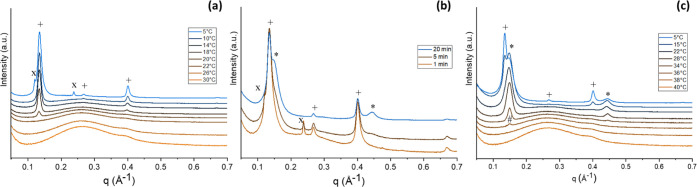
MF1 SAXS temperature profile patterns: slow cooling at −0.5 °C/min (a); holding at 5 °C (b); heating at 0.5 °C/min (c). Patterns were stacked at constant offset for better readability. Peaks belonging to the same polymorph are indicated with the same symbol: (+), (x) α1(2L) and α2(2L); (*) β′(2L), (#) β(2L).

The crystallization behavior and polymorphic outcome of MF1 were investigated in situ, with SAXS/WAXS; in particular, we focused on the identification and characterization of different polymorphs and on the detection and analysis of nucleation events, which from now on will be referred to with the more general term “nucleation”. During the first crystallization profile performed at a cooling rate of −0.5 °C/min (Figure 6a), a peak at 0.133 Å^–1^ (*d* = 47 Å) appeared first at 20 °C. Considering that MF1 is mainly composed of TAGs with an average length of 17.2 C atoms, this peak was assigned to a α(2L) form. At lower temperature (7–8 °C), another peak at a slightly higher *d*-spacing appeared (*q* = 0.121 Å^–1^, *d* = 52 Å), suggesting the presence of another metastable 2L polymorph, starting to disappear at around 20 °C, upon heating (Figure 6a). The higher value of *d*-spacing means that this second form originated from a fraction of MF1 containing TAGs with longer fatty acid chains compared to the α form previously nucleated, which are immiscible with those forming the first α(2L) polymorph. Moreover, this polymorph nucleated and melted at a lower temperature compared to the first α(2L) form (7 and 20 °C, respectively), which might be related to the higher degree of unsaturation of its forming TAGs (e.g., OOP and OOS).^35^ These two immiscible 2L polymorphs will be named α_1_ (*d* = 47 Å) and α_2_ (*d* = 53 Å) from now on, for clarity.

After a few minutes of holding at 5 °C, the peak at *q* = 0.121 Å^–1^ (α_2_) disappeared, while a peak at *q* = 0.149 Å^–1^ (associated with the β′(2L) observed at room temperature) started to appear, indicating a polymorphic transformation from α to β′. In Figure 6b, the presence of the β′(2L) form is more evident in the third-order reflection region, where the peaks of these two polymorphs do not overlap.

Upon heating at 0.5 °C/min, the remaining α polymorph melted completely at 22–23 °C, whereas the peak of the β′(2L) remained visible up to 36 °C.^46^

MF1 was then cooled again down to 5 °C, but this time with a faster cooling rate (−5 °C/min), as shown in Supporting Information file, Section 2, Figure 2.3. The results showed no significant difference compared with the slower cooling experiment, in terms of polymorphic behavior. The α_1_ polymorph nucleated first at 17 °C, and then at 5 °C, the double chain α_2_ appeared. Both forms nucleated at lower temperatures compared to the previous experiment. The difference in terms of crystallization temperatures in the two cooling processes is explained by the slower cooling rates, providing more time for critical size nuclei to form; thus, slower cooling rates lead to nucleation at lower degrees of undercooling.^19^

**Figure 7:**
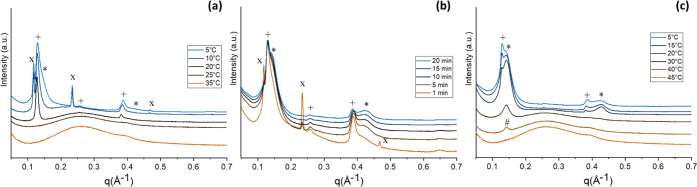
MF2 SAXS temperature profile patterns: slow cooling at −0.5 °C/min (a); holding at 5 °C (b); heating at 0.5 °C/min (c). Patterns were stacked at constant offset for better readability. Peaks belonging to the same polymorph are indicated with the same symbol: (+), (x) α1(2L) and α2(2L); (*) β′(2L), (#) β(2L).

Sample MF2 was also recrystallized at a slow cooling rate of −0.5 °C/min (Figure 7a). The first peak (*q* = 0.129 Å^–1^, *d* = 48.6 Å) was observed at around 23 °C, and it grew together with the second- and third-order reflections of the same polymorph, identified as the α_1_ form, similarly to MF1. During the cooling profile, another form (*q* = 0.117 Å^–1^, *d* = 53.5 Å) crystallized between 13 and 12 °C; this is a α_2_ form similarly to the one observed for MF1. In the last part of the cooling process, before reaching the target temperature of 5 °C, the peaks of both of these polymorphs continued to increase in intensity, while shoulders from a more stable polymorph started to appear. Indeed, during the following holding profile (Figure 7b), where the temperature was kept constant at 5 °C, these third form shoulders grew into peaks (first-order peak *q* = 0.138 Å^–1^, *d* = 45.5 Å) related to a more compact structure β′(2L) form.^39^ The Supporting Information file, Section 3, Figures 1–6 report graphs describing the linear relationship, typical of lamellar phases, between the q values of peaks belonging to the same polymorph and the h Miller indexes of consecutive parallel planes. The slope of these lines corresponds to the characteristic lamellar *d*-spacing of the polymorph studied. Regarding the heating profile (Figure 7c) α_1_ was observed melting at 25 °C, while α_2_ was no longer visible, possibly covered by the β′(2L) polymorph intense peaks, which were still partly visible above 40 °C. The sample was completely melted at 45 °C.

MF2 then underwent a second and faster cooling profile (Supporting Information file, Section 2, Figure 2.4): the type and number of polymorphs identified were the same as those in the experiments at lower cooling rates, but some differences were observed. First, nucleation happened at 18 °C, when peaks characteristic of the α_1_ form began to appear, while the α_2_ form started to form again at lower temperatures, becoming visible around 8 °C. At 0 °C, both α forms were still present and a third trilayered form appeared, characterized by a weak first-order reflection peak. This polymorph is consistent with a γ(3L) polymorph (*d* = 75 Å), described previously by Mykhaylyk and Hamley for SOS.^40^ This is also supported by the low melting point of this polymorph, which completely melted before 20 °C, and it is related to the presence of SOS in MF2.

**Figure 8:**
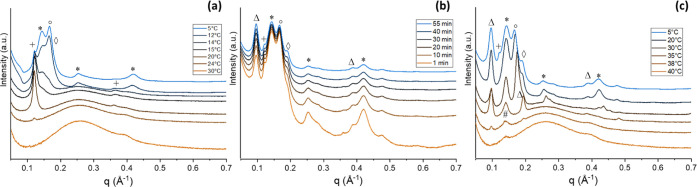
MF3 SAXS temperature profile patterns: slow cooling at −0.5 °C/min (a); holding at 5 °C (b); heating at 0.5 °C/min (c). Patterns were stacked at constant offset for better readability. Peaks belonging to the same polymorph are indicated with the same symbol: (+) α(2L), (*) β′(2L), (#) β(2L), (°) γ(3L), (◊) β′(3L).

MF3 SAXS/WAXS patterns during cooling experiments are shown in Figure 8. During the first cooling cycle at −0.5 °C/min, a peak at *q* = 0.120 Å^–1^ started to form at 24 °C (*d* = 52.1 Å), which was associated with a α(2L) form. This structure has a longer *d*-spacing (52.3 Å) than the α_1_(2L) form of MF1 (*d* = 47 Å). This may be explained by the presence of longer fatty acids in the TAGs forming MF3 compared to MF1. Using the equation reported in the literature (*d* = 2(1.27*N*_c_) + 8)^19,47^ to estimate the *d*-spacing of a mixture of TAGs with an average chain length of 17.9 carbon atoms, a theoretical value of 53.4 Å is obtained, which is consistent with the experimental value of around 52 Å.

With further cooling, at 15 °C, a shoulder at higher values of q started to form. Three separate forms became distinguishable at such temperature: a β′(2L) (*q* = 0.142 Å^–1^, *d* = 44.1 Å), already present at 20 °C and two polymorphs with overlapping peaks at *q* = 0.166 Å^–1^ and *q* = 0.18 Å^–1^ (Figure 8a). These peaks are the second-order reflections of two 3L structures with *d*-spacings of about 75 and 69 Å. These long-range *d*-spacings are likely belonging to γ (3L) and β′(3L) polymorphs, respectively, which are typical of SOS.^40^ Mykhaylyk and Hamley^40^ described them as intermediate forms, in terms of stability, between α(2L) and β(3L); these polymorphs were also reported in other studies.^47−49^ After 15 min of holding MF3 at 5 °C, the peak at *q* = 0.095 Å^–1^ (*d* = 65.9 Å), corresponding to the equilibrium β(3L) polymorph, became visible (Figure 8b). This confirms that in MF3, the transformation to the most stable polymorph occurred more rapidly compared to MF2, which did not show signs of β(3L) form during the cooling and isothermal holding processes. Upon heating at 0.5 °C/min, the least stable α(2L) polymorph disappeared first at 20 °C (Figure 8c). In the temperature range of 10–30 °C, the intensity of the γ(3L) polymorph peaks progressively decreased, probably because this form started to convert into the more stable β(3L) form. The β′(3L) form melted between 32 and 34 °C, whereas the β(3L) peak persisted up to 38 °C. A peak corresponding to a 2L polymorph was still visible at 40 °C. This is probably a β(2L) polymorph generated by a fraction of high melting TAGs (e.g., trisaturated such as SSS), which could not be identified before because of the overlapping with the peaks of the more unstable forms.^50,51^

After the sample was fully melted upon the first crystallization cycle, another one was performed with a faster cooling rate of −5 °C/min (see Supporting Information, Section 2.5, Figure 5e). The results in this case were similar to MF1: the α_1_(2L) polymorph (*d* = 52.3 Å) nucleated first, between 23 and 18 °C, at a lower temperature compared to the previous cycle. Then a α_2_ form, with a higher *d* spacing (*d* = 56 Å), not observed in the previous crystallization cycle, appeared between 13 and 8 °C. This α polymorph remained during the holding period at 5 °C, when the β′(2L) characteristic first-order peak started to be evident. In this case, neither the γ nor the β′(3L) polymorphs were observed.

**Figure 9:**
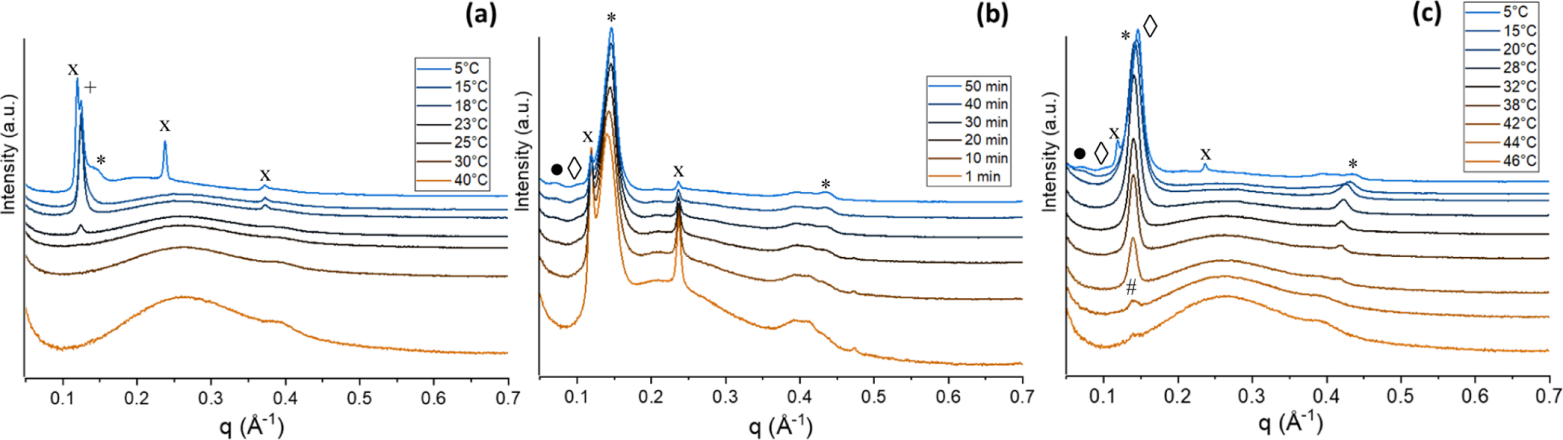
MF4 SAXS temperature profile patterns: slow cooling at −0.5 °C/min (a); holding at 5 °C (b); heating at 0.5 °C/min (c). Patterns were stacked at constant offset for better readability. Peaks belonging to the same polymorph are indicated with the same symbol: (+), (x) α1(2L) and α2(2L); (*) β′(2L), (#) β(2L), (◊) β′(3L), (●) α(3L).

For MF4, a first cooling ramp led to the formation of numerous peaks, as shown in Figure 9a. The first peak (*q* = 0.124 Å^–1^, *d* = 50.5 Å) became visible at around 24 °C, and it was attributed to a first α_1_ form, which kept growing until a second α_2_ form appeared at 10.9 °C (*q* = 0.019 Å^–1^, *d* = 52.8 Å). At 10.9 °C, a shoulder started to be visible on the right of the first peak at *q* = 0.07 Å^–1^. However, it did not fully develop during the cooling ramp and the following 10 min holding; thus, MF4 was then kept at 5 °C for longer than the other samples (around 50 min) to enable crystallization.

In Figure 9b, it is possible to observe changes in the number and type of polymorphs during this prolonged period of holding. The α_1_ form crystallized at higher temperature disappeared due to its instability. The α_2_ form decreased as well, even though its peaks were still observed longer compared to those of α_1_. On the other hand, the shoulders that were observed at the end of the slow cooling ramp grew into two overlapping peaks centered at around *q* = 0.14 Å^–1^. At the end of the holding time, four different polymorphs could be distinguished. A first one was the α_2_ form originated during cooling, while overlapping peaks from an unstable 3L polymorph (with first order peak at *q* of around 0.08 Å^–1^) and a more stable β(2L) developed after 15 min at 5 °C. At 25 °C peaks from another metastable 3L forms are visible, with the first peak still weaker than the second one. The *d*-spacing for all these polymorphs is summarized in Table 6. Due to their low concentration and the presence of other forms, a definite polymorph identification is not possible; however, considering the *d*-spacing, melting points, and the composition of MF4, it is likely that the one with a shorter *d*-spacing of 61 Å is a β′(3L)^52^ rich in OOS, whereas the one with a *d*-spacing of 87 Å might be an α(3L) related to the presence of SSO.^53^

**Table 6:** *d*-Spacings in Å for All Polymorphs Detected in Each Sample during Cooling Profiels and Isothermal Steps (20 °C or 5 °C)^a^

polymorph	MF1	MF2	MF3	MF4	MF	CB
α(2L)	47, 53	49, 54	51, 56	51, 53	46	49, 54
α(3L)	n.d.	n.d.	n.d.	87	59, 72	n.d.
β′(2L)	42	46	45	n.d.	41	44
β′(3L)	n.d.	n.d.	71	61	66	n.d.
γ(3L)	n.d.	traces	75	n.d.	n.d.	n.d.
β(3L)	n.d.	70	65	n.d.	n.d.	64
β(2L)	n.d.	43, 47	45	44, 45	n.d.	43, 44, 50

a N.d. = not detected.

The following heating process provided a better understanding of the type and number of polymorphs formed at 5 °C. In Figure 9c, the peaks of the two 3L polymorphs were already decreased between 10 and 13 °C and completely melted before 25 °C. Peaks from the α forms fully disappeared before 13 °C, coherently with the instability typical of this polymorph. At 22 °C, only the most stable β(2L) form was still visible and kept growing. At 40 °C, the last trace of a first-order β(2L) peak was the only one still detectable (Figure 9b). As mentioned earlier, β(2L) polymorphs are characteristic of high melting TAGs (e.g., trisaturated TAGs, molecular compounds). The dynamic study of crystallization was repeated by applying a faster cooling rate. At 5 °C, only the two α forms were visible, and their crystallization temperatures were lowered by several degrees compared to the slower cooling profile, as the first peak was observed at 20 °C instead of 24 °C and the second form peaks appeared at 10 °C.

A summary of all of the polymorphs detected in the MFAs, MF, and CB is shown in Table 6. All samples behave differently, but MF2 and CB are more similar in terms of number and type of polymorphs. Indeed, these two samples have a similar amount of SOS and a significant amount of POP and POS (this TAG is higher in CB than MF2).

### 20% Blends of MF and MF*n* in CB

To investigate how the addition of MF or MF*n* to CB affects its crystallization behavior, mixtures with 20% in mass of MF, MF1, MF2, MF3, and MF4 were prepared and analyzed at room temperature after 1 week with both PXRD and SAXS.

**Figure 10:**
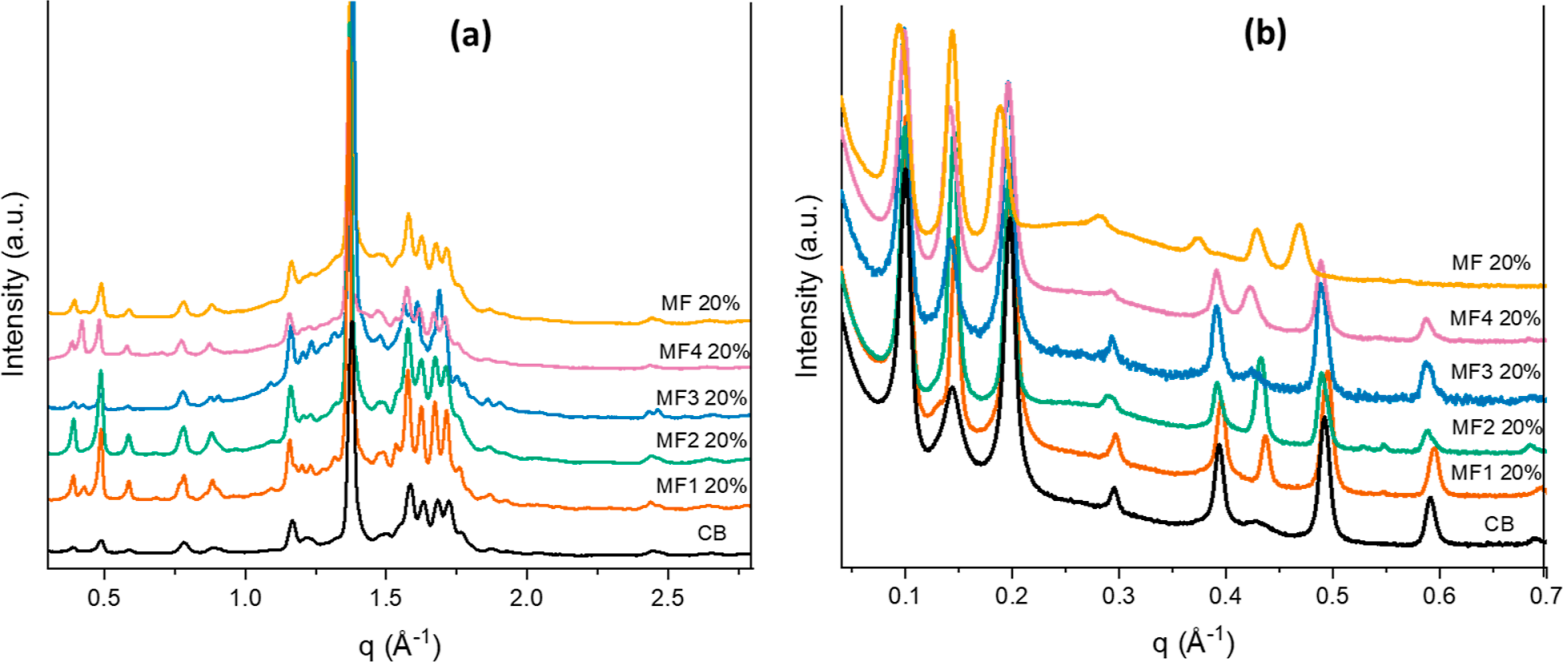
(a) PXRD diffractogram and (b) SAXS patterns collected at 20 °C for CB, MF*n* 20%, and MF 20% mixture: CB in black, MF1 20% in brown, MF2 20% in green, MF3 20% in blue, MF4 20% in pink, and MF 20% in orange. The PXRD patterns show a prevalent, similar β(*V*) polymorph in all samples. The presence of additional β polymorphic forms with 2L stacking is visible in the SAXS patterns for all samples. For clarity, the dotted lines indicate the position of the SAXS peak of the β(*V*) form of CB.

PXRD diffractograms of the mixtures did not show significant differences compared to pure CB. The wide-angle patterns reported in Figure 10a showed a sharp peak with *d* = 4.6 Å and four less intense peaks at higher values of *q*. The values of *d*-spacing of these minor peaks were identical to CB in all the blends (*d* = 3.97 Å, 3.87 Å, 3.74 Å, and 3.64 Å) and they were all indicative of the presence of the β(*V*) polymorph of CB.

In the SAXS/WAXS pattern of the MF 20% blend (Figure 10b), it can be noticed that the intensity of the β(*V*) peaks was lower compared to CB and the other mixtures, with higher *d*-spacing (MF 20% *d* = 66.1 Å CB *d* = 63.9 Å) indicating differences in the stacking on the TAGs. A second β(2L) form is also visible for this sample, with a *d*-spacing of 43.8 Å. When the MF 20% blend was heated, the β(3L) form melted between 32 and 34 °C, earlier than the β(2L), whose peak persisted up to 38 °C. A melting temperature of about 33 °C is consistent with the values reported in the literature for the β(*V*) form of milk chocolate.^54^

As depicted in Figure 10b, at 20 °C, the mixtures MF1 20% (brown) MF2 20% (green), and MF3 20% (blue) showed a similar SAXS pattern to the one of pure CB (black). The peaks of the β(3L) polymorph are almost superimposable with those of CB, apart from some shifts in the higher reflections. The peaks of the β(2L) forms seem different, as they are associated with the presence of different minor TAGs in each of the MF*n*s.

Compared to pure CB, in all mixtures at 20% MF or MF*n* (Figure 10a), it is possible to notice the presence of a broad WAXS peak in the region 1–2 Å^–1^, related to the scattering of the liquid phase. This is particularly visible for MF, MF3, and MF4, which present the lowest SFC of all the tested samples.

Regarding the SAXS/WAXS experiments performed with temperature profiles, it was observed that the number and types of polymorphs nucleated in MF 20% and MF*n* 20% were the same as CB.

Indeed, SAXS/WAXS patterns of all mixtures resembled very much CB patterns in terms of polymorphic contribution and crystallization/melting behavior, with variations on melting and nucleation temperatures in a range of ±2 °C. The specific composition of each MF*n* did not bring to the blend any detectable difference in terms of number and types of polymorphs nucleated compared to CB.

**Figure 11:**
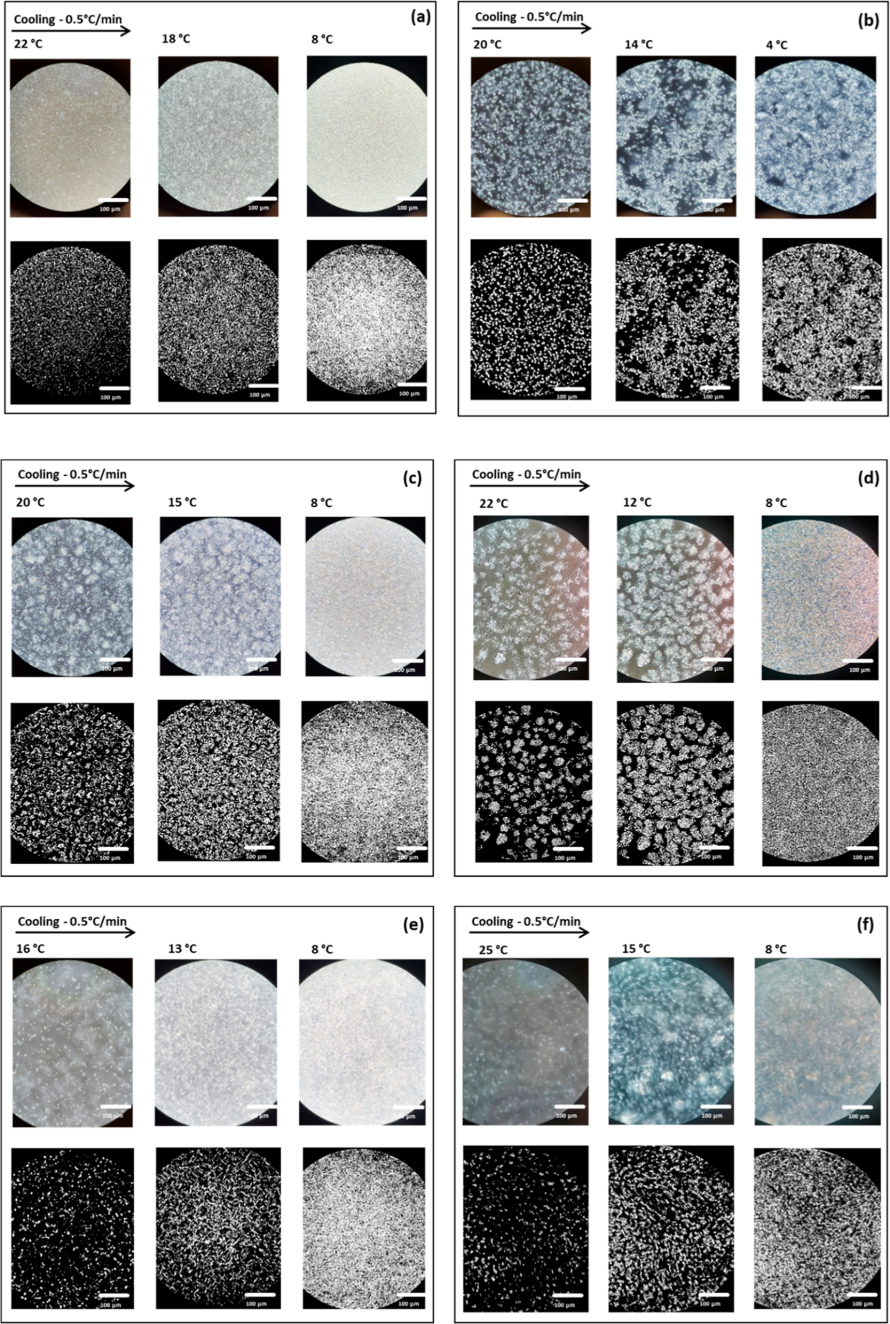
CB (a) and MF (b) video frames recorded during a crystallization experiment (cooling at −0.5 °C/min) with the HS-PLM (Linkam) for CB (a), MF (b), MF1 (c), MF2 (d), MF3 (e), MF4 (f), and their binarized version.

SAXS/WAXS patterns collected during cooling, holding, and heating profiles for all the mixtures are reported in Supporting Information, Section 2.7, Figures 7–11.

### HS-PLM Analysis of the Crystalline Microstructure and Crystallization

**Figure 12:**
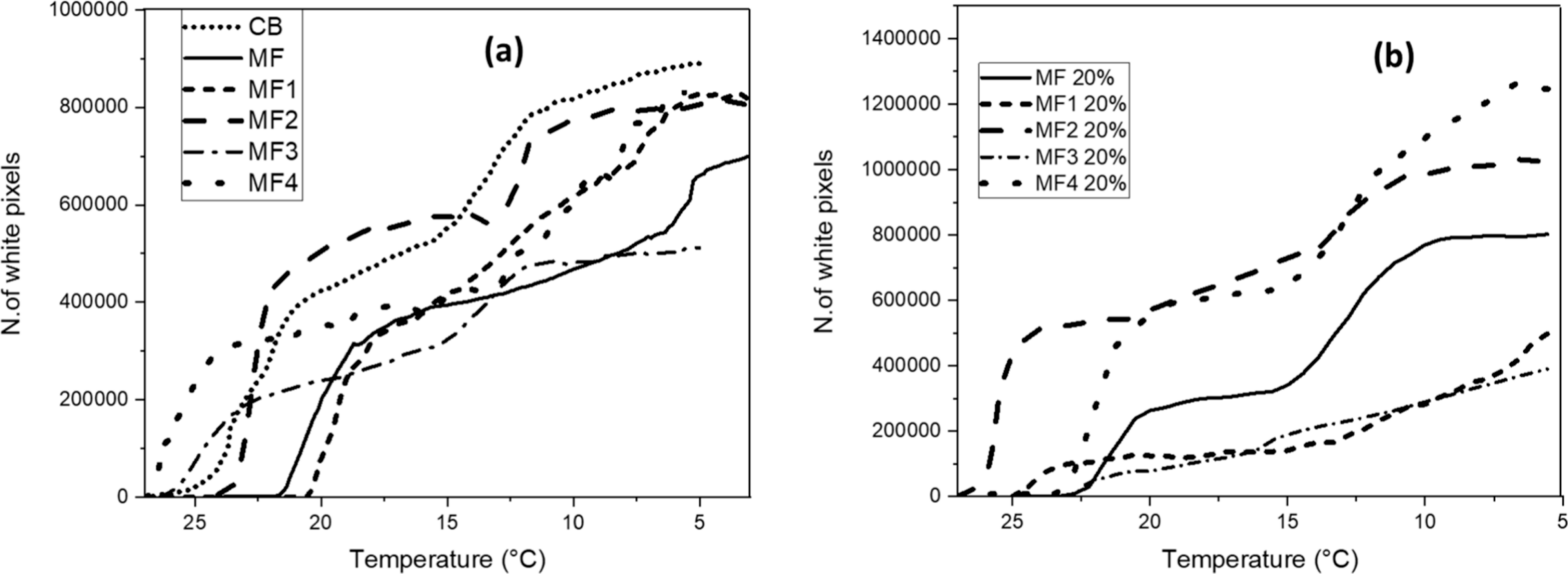
(a,b) Plot depicting the results obtained for the HS-PLM experiment at −0.5 °C/min. Frames were extracted and processed every 30 s (one image every 0.25 °C). White pixels were counted using a MATLAB script and exported as graph with the number of pixels expressed in function of the temperature (°C). Pure compounds are shown in figure (a), whereas mixtures at 20% MF or MF*n* are reported in figure (b).

Hot stage polarized light microscopy (HS-PLM) was used to study the microstructure of the crystallized fat mixtures. It is worth pointing out that PLM is not a bulk technique, and only the surface of a small portion of the sample is observed. Figure 11a shows frames taken during the crystallization experiment performed at −0.5 °C/min and their corresponding binarization. Graphs reporting the number of white pixels as a function of time during the cooling crystallization experiment at a rate of −0.5 °C/min are shown in Figure 12. Two separate crystallization experiments, at different cooling rates, were performed in order to evaluate the effect of the cooling rate on the microstructure of the crystalline network formed and to be able to qualitatively compare the PLM data with the results of the SAXS experiments. In fact, due to the different sample volumes and geometries of the sample holders (e.g., different heat transfer coefficients), it is not possible to directly relate the results obtained with SAXS/WAXS with those from PLM analysis or DSC, even when comparable cooling and heating rates were applied. However, it is still possible to make some qualitative observation on the number and type of polymorphs appearing during sample cooling, especially considering that all techniques (PLM, SAXS/WAXS, and DSC) showed consistent results for the different cooling rates applied.

The images of Figure 11 show that for all samples studied crystals nucleated above 20 °C and then grew as the temperature was decreased. Toward the end of the cooling profiles, it is possible to notice a decrease in size of the crystalline aggregates formed in the early stages of cooling and the appearance of new smaller ones. This partial melting and recrystallization is typical of a polymorphic transformation, in agreement with what was observed in the SAXS/WAXS experiments.

This behavior is also observable in most of the trends of the white pixel numbers as a function of temperature (Figure 12) where a first sharp increase in pixels is associated with a first nucleation event, then there is a region of slower increase where crystals grow, and finally a second sharp increase in white pixels is associated with a second nucleation event due to the formation of different, more stable polymorphs (e.g., polymorphic transformation) that continues to grow. Table 7 shows the rate of increase in white pixels per °C in different regions of the cooling profile. It is worth noticing that in some cases, it was not possible to discriminate between nucleation and growth and nucleation and polymorphic transformation; in such cases only one rate per both regions was calculated.

**Table 7:** Quantitative Analysis of the PLM Trends and Rate of White Pixels Increase Per °C in the Different Regions of the Cooling Profile (as Shown in Figure 12)

	rate of white pixel increase (white pixels/°C)			
sample	first nucleation event	growth	polymorphic transformation	growth
CB	9.8 × 10^4^	2.5 × 10^4^	7.7 × 10^4^	1.5 × 10^4^
MF	1.2 × 10^5^	1.4 × 10^4^	6.5 × 10^4^	5.5 × 10^3^
MF1	1.6 × 10^5^	3.8 × 10^4^	7.0 × 10^4^	5.8 × 10^2^
MF2	2.9 × 10^5^	1.6 × 10^4^	9.3 × 10^4^	7.1 × 10^3^
MF3	7.3 × 10^4^	1.5 × 10^4^	5.2 × 10^4^	5.2 × 10^3^
MF4	9.6 × 10^4^	1.3 × 10^4^	5.1 × 10^4^	
MF 20%	1.2 × 10^5^	1.4 × 10^4^	1.0 × 10^4^	3.0 × 10^3^
MF1 20%	6.5 × 10^4^	4.9 × 10^3^	3.7 × 10^4^	
MF2 20%	3.1 × 10^4^		7.0 × 10^4^	4.6 × 10^3^
MF3 20%	1.41 × 10^4^	1.39 × 10^4^	5.6 × 10^4^	2.1 × 10^4^
MF4 20%	2.6 × 10^5^	2.6 × 10^4^	1.1 × 10^5^	5.3 × 10^4^

The PLM data related to CB [Figure 12a (dotted line)] showed a rapid increase of the number of white pixels at about 23 °C, corresponding to nucleation of the α(2L) crystals, and then the curve presented a constant slope related to growth of this form, up to 15 °C. At this point, the curve became steeper, indicating the nucleation and then growth of another form, most likely the β′(2L) observed at 14 °C in the SAXS/WAXS experiments.

In the MF curve, the first nucleation event occurred at 22 °C. Then, the curve slope increased slightly at about 14 °C, when crystalline aggregates with a more elongated shape started to develop, as shown in Figure 11b. It is possible that the first crystals that nucleated were those of the α(2L) form, whereas the fraction that crystallized at 14 °C were α(3L) crystals, which is consistent with what was observed by Pratama et al.^19,54^ Below 10 °C, the slope of the curve increased more drastically, as the crystal aggregates seemed to change shape (Figure 11b). In the SAXS/WAXS patterns, a transformation from α(3L) to β′(3L) was observed below 10 °C during the crystallization at −0.5 °C/min (Supporting Information file, Section 1.2, Figure 12b). It is possible that the increase in slope of the curve of white pixels refers to this polymorphic transformation.

In the MF1 curve [Figure 12a (short dashed line)] after the nucleation of the α_1_ polymorph at 20 °C, the number of white pixels increased linearly, with a steeper slope from 8 °C. This last increase in the number of white pixels possibly refers to the crystallization of the α_2_ polymorph.

MF2 [Figure 12a (long dashed lines)] underwent two sharp crystallization steps at ∼22 and ∼12 °C, likely related to a polymorphic transformation. Indeed, it is possible to observe it in Figure 11d; MF2 first showed few spherical agglomerates at 22 °C, increasing in number and in size in the second nucleation step at around 12 °C, until a compact crystal network was obtained.

The α_1_ form of MF3 nucleated at 26 °C. After a first rapid increase, the curve related to the number of white pixels [Figure 12a (dashed and dotted line)] showed a less steep slope up to 16 °C. At this temperature, a second rapid increase in number of white pixels was observed. At 15 °C, a β′(2L) polymorph appeared during the SAXS/WAXS analysis with the same cooling rate of −0.5 °C/min. Thus, it is possible that the change in slope at 16 °C indicates the transformation from α(2L) to β′(2L). No visible differences were noticed later in the shape of the crystalline aggregates, as shown in Figure 11e.

The MF4 [Figure 12a (far apart dashed lines)] sample started crystallizing above 25 °C and behaved slightly differently from the previous ones. Elongated crystalline aggregates formed and initially grew without any spherical structure formation, as is possible to notice in Figure 11e. Later, it was possible to see wider spherical agglomerates in the background, coexisting with the needles. This suggests the coexistence of different immiscible crystal structures forming at different temperatures (possibly the α, or β′ and a more stable β form).

**Figure 13:**
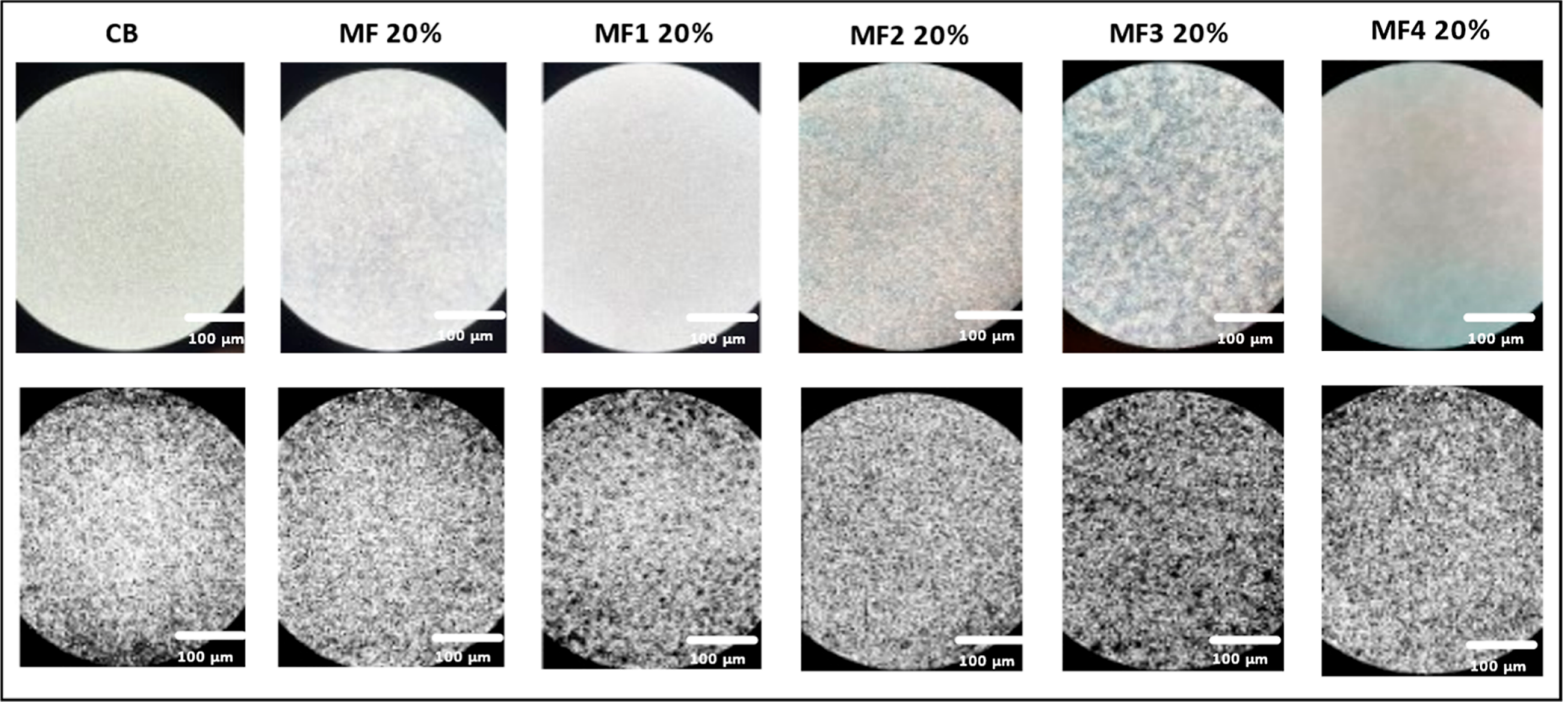
Video frames taken at the end of the crystallization experiment (−0.5 °C/min) for CB, MF 20%, and all MF*n* 20% mixture in comparison with each other.

Figure 12b reports the trends observed for the mixtures of MF*n* with CB. In terms of the crystalline microstructure, no significant difference from pure CB was observed, and a homogeneous distribution of crystals within the field of view was observed for all samples when crystallization was complete. Figure 13 shows frames taken at the end of the crystallization experiment (−0.5 °C/min) for CB and each 20% mixture for the sake of comparison. It is clearly noticeable that the shape of crystalline aggregates in the mixtures resembles the ones of CB. The main difference was observed after the binarization process and the data plot, which shows that the total number of white pixels per unit area in the case of MF1 20% and MF3 20% was lower compared to the other blends, possibly due to different crystallization kinetics. MF2 20% and MF4 20% crystallization profiles appeared instead to be closer to MF 20%, both in number of pixels and in the white pixels trends.

### Differential Scanning Calorimetry

The main DSC events with onsets and offsets observed during the cooling/heating ramp at ± 2 °C/min are reported schematically in Table 8 for CB, MF, and MF*n*. All DSC curves are collected in the Supporting Information file, Section 4. For all samples, the cooling rate applied did not seem to affect the number of thermal events observed, but slight changes in the onset temperature were detected, as observed also during the SAXS/WAXS experiments. Due to differences in the sample volumes and holder geometries, it is not possible to compare directly the DSC signal with the results obtained with X-ray scattering. Nevertheless, especially considering the small effect of cooling rate on the DSC thermograms, we can still relate qualitatively the number of DSC peaks and their melting range to the type and relative stability of the polymorphs observed via X-ray scattering.

**Table 8:** DSC Onset and Offset Ranges of the Main Thermal Events for CB, MF, and MF*n*

sample	CB	MF	MF1	MF2	MF3	MF4
cooling (−2 °C/min)	21–17 °C	20–15 °C	19–15 °C	22–18 °C	24–19 °C	24–17 °C
	15–8 °C	16–12 °C	10–0 °C	15–5 °C	15–5 °C	15–8 °C
	5–0 °C	12–0 °C	5 °C–(−)2 °C	3–2 °C	3–2 °C	5–2 °C
heating (2 °C/min)	10–30 °C	0–11 °C	5–22 °C	2–10 °C	–5–5 °C	4–6 °C
		12–17 °C	22–34 °C	10–28 °C	5–7 °C	10–27 °C
		17–36 °C		28–35 °C	10–25 °C	27–42 °C
					25–38 °C	

The DSC curves obtained via cooling of CB showed two main exothermic peaks and a smaller peak at lower temperature (at around 2 °C). These peaks can be associated with the appearance of the α_1_, β′, and α_2_ polymorphs, similarly to what was observed in the SAXS/WAXS experiments.

The first exothermic event started at 21 °C in the slower cooling profile and at 20 °C in the faster one. During the heating ramp, three endothermic events occurred, although their peaks seemed to be partly overlapping. These events correspond to the melting of the α_1_ and α_2_ polymorphs, subsequently followed by the melting of more stable polymorphs, possibly the β′ and β(2L) forms observed with X-ray scattering.

For MF, it is possible to distinguish two major exothermic events during both the cooling ramps. The first started at about 20 °C in the slower crystallization profile and at 18 °C in the faster one. This first exothermic event may be associated with the nucleation of the MF α(2L) form. The second major peak onset is recorded at 12 °C in the cooling at −2 °C/min and at a slightly lower temperature for the faster cooling profile. This exothermic event may refer to the crystallization of one of the less stable α1(3L) forms observed in the SAXS/WAXS patterns (Supporting Information file, Section 2, Figure 2.2). Between the two major exothermic events, other smaller and less defined, partly overlapping peaks appeared in the range 16–12 °C; these might be attributed to the formation of the other metastable polymorphs observed in the SAXS/WAXS experiments, particularly the α2(3L) and the β′(2L).

The melting curve of MF does not have a clear baseline, and the first peak in the range 0–11 °C is not easy to resolve. Additionally, exothermic events centered at 11–12 and 17 °C can be detected corresponding to polymorphic transformation of the α(2L), α(3L) and β′(3L) forms into the prevalent β′(2L) observed at ambient temperature. The last broad endothermic peak between 20 and 36 °C is associated with the melting of this β′(2L) form, in agreement with the SAXS/WAXS analysis.

In the DSC curves obtained during cooling of MF1, two main exothermic peaks are visible, together with a smaller peak at subzero temperature. Both major exothermic peaks are possibly associated with the crystallization of the α_1_ and α_2_ polymorphs, as observed in the SAXS/WAXS. The first polymorph nucleated at about 19–18 °C depending on the cooling rate applied, whereas the second peak started at 10 °C. A weak peak at temperatures below zero (−2 to −5 °C) is visible, and it probably represents the crystallization of a low melting fraction of MF1 (e.g., containing polyunsaturated TAGs as shown in Table 4). The melting curve shows several overlapping events in the range 5–22 °C. Eventually, these enlarged overlapping events could be attributed to the melting of the two α forms and possibly to the polymorphic transformation into the more stable β′(2L) and β(2L), whose melting is observed between 23 and 32 °C.

The thermograms of MF2 are consistent with the SAXS/WAXS data obtained during cooling. The first exothermic event occurred in the range 22–18 °C, while a second one can be observed at around 15 °C, ending at 5 °C; these events are associated with the formation of the α_1_, α_2_, and β′(2L) polymorphs observed in the dynamic SAXS/WAXS experiments. A smaller peak is also detected at around 0 °C; this might be associated with the crystallization of the lower melting fraction of MF2, similarly to what was observed in MF1.

Upon heating, a broad melting range is observed, with three different macro regions identified: from 2 to 11 °C, from 12 to 28 °C, and from 29 to 35 °C. The presence of multiple peaks, especially between 12 and 28 °C, indicates possible polymorphic transformation during the heating ramp. While the lower melting peak can be associated with low melting and metastable polymorphs (e.g., α_2_), the central meting region corresponds to the melting of α_2_ and β′(2L), whereas the high melting peak is possibly associated with the fusion of the more stable β(2L) and β(3L) polymorphs observed at ambient temperature.

The first exothermic event in the DSC curve obtained by cooling MF3 started at about 24 °C for the slower cooling rate and at 22 °C for the faster cooling profile. These temperatures are in accordance with the SAXS/WAXS patterns for the α(2L) form crystallization range. The second exothermic peak started at 15 °C in both cooling ramps. The onset temperature of this second exothermic event could suggest the crystallization of the β′(2L) form as observed in the SAXS/WAXS experiments. Two more nucleation events are detected during cooling, at around 5–6 °C [possibly γ(3L) and β′(3L), as was detected in the SAXS/WAXS, which are observed to melt at around 7 °C upon heating] and below zero (this might be the crystallization of the low melting fraction of MF3, normally liquid at ambient temperature, which subsequently melts during heating at around −4 °C). The presence of these low melting fractions, composed of polyunsaturated TAGs (as evident in Table 5), might have an enhancing effect on the subsequent polymorphic transformation, as shown in previous literature.^55^

A distinct exothermic event, which started at 10 °C and ended at about 25 °C, occurred during heating and is indicative of a polymorphic transition, perhaps from the α(2L) forms and the β′(2L) polymorph to the more stable β(2L) forms and β(3L) polymorph observed at ambient temperature. A broad peak was also visible at high temperatures (up to 38 °C), probably indicating the melting of these stable forms.

The MF4 sample showed a complex DSC profile, similar to MF. Indeed, both samples are characterized by a wide range of TAGs and an abundant low melting TAG fraction that remains liquid in the temperature range of the analysis. During cooling, the first step of crystallization appeared in the range 24–17 °C, which is in accordance with the SAXS/WAXS data for the nucleation of the α_1_ and α_2_ forms. A second exothermic event characterized by a broad peak started at 15 °C in both cooling profiles. Possibly in this range of temperature we observed the nucleation of the β′(2L) and the two α(3L) polymorphs, as seen during the SAXS/WAXS experiments. A smaller exothermic peak is visible at 2–3 °C, similarly to what is observed in the other samples (e.g., crystallization of low melting TAGs).

The thermogram recorded during heating of this sample is complex and characterized by both endothermic and exothermic peaks. The last ones are associated with polymorphic transitions from the more metastable forms (α and β′) to the more stable β(2L) polymorphs observed at ambient temperature. This is also confirmed by the high temperature of the end of melting, which is around 42 °C.

Despite differences in the DSC of the pure components and in the polymorphic landscape, the thermal profiles of the MF*n* 20% blends are remarkably similar to that of the mixture MF 20%: Table 9 reports the temperatures and ranges of the endothermic and exothermic peaks observed. Nevertheless, some differences were noticeable especially in MF3 20% and MF4 20%, which possess a broader melting range compared to MF 20% (e.g., MF 20% = 5–25 °C, MF3 20% = 5–29 °C, MF4 20% = 4–28 °C).

**Table 9:** DSC Onset and Offset Ranges of the Thermal Events for MF 20% and MF*n* 20%

sample	MF 20%	MF1 20%	MF2 20%	MF3 20%	MF4 20%
cooling (−2 °C/min)	21–16 °C	22–17 °C	20–16 °C	22–17 °C	21–16 °C
	15–8 °C	16–8 °C	16–7 °C	17–8 °C	15–7 °C
	2 °C–(−)2 °C	5–0 °C	3 °C–(−)2 °C	5–0 °C	3 °C–(−)1 °C
heating (2 °C/min)	5–25 °C	7–30 °C	8–28 °C	5–30 °C	4–28 °C

## Conclusions

This work investigated the crystallization behavior of four plant-based TAG mixtures (MF*n*) to be used in vegan milk chocolate and their 20% w/w blends with a sample of cocoa butter of Ghanian origin. The effectiveness of these mixtures of fats to emulate the crystallization behavior of milk fat when mixed with cocoa butter was investigated. The TAG composition of each mixture was measured and compared to that of milk fat and cocoa butter to understand whether the thermal behavior and crystalline polymorphs of milk fat blends can be replicated with fat mixtures with different TAG composition. All pure samples presented different crystallization behaviors; in POP-rich sample MF1, the polymorph detected at ambient temperature was a β′(2L), indicating slow kinetic of transformation toward the more stable β polymorph (e.g., this was the only sample still presenting a metastable polymorph after several days of isothermal equilibration). Only α(2L) polymorphs were recorded during the dynamic experiments. Sample MF2 presented a similar polymorphic behavior to CB, with a β(3L) and two immiscible β(2L) forms stable at ambient conditions; this is due to the high percentage of POP and SOS in this sample, together with 2L crystallizing TAGs such as trisaturated or molecular compound forming ones.

In MF2, multiple metastable 2L forms appeared during the cooling profile (two α and a β′); this is consistent with the behavior of CB. Traces of a γ(3L) polymorph, typical of SOS, were also detected for MF2.

Sample MF3, rich in SOS, was similar to CB at ambient, stable conditions, whereas more metastable phases were detected upon cooling from melt: two α(2L) and a β′(2L), a γ(3L), and a β′(3L). Finally, sample MF4 showed only stable β(2L) polymorphs at ambient conditions, likely resulting from molecular compound forming TAGs pairs (e.g., OOS/SOS and OSO/SOS) and/or trisaturated TAGs. Upon cooling, 3L forms with *d*-spacing consistent with the *d*-spacing of the OOS and SSO metastable polymorphs appeared. Despite these differences, which are also determining the different SFCs of these samples, when mixed with CB in a 20/80 w/w ratio, all MFAs presented a similar polymorphic landscape in terms of number, type, and melting range to the MF/CB mixture (within ±3 °C for both the onset and end melting temperatures). In fact, a similar β(3L) polymorph (in terms of *d*-spacing and melting point) was detected in all mixtures at equilibrium, with small differences in the number and *d*-spacing of the immiscible, high melting β(2L) forms still present at ambient conditions. It is worth noticing that while the main chocolate properties are due to the features of the β(3L) polymorphs, the presence of several, stable high melting polymorphs might affect tempering processes as well as slower phenomena such as fat blooming and oil migration. A careful study of the polymorphic landscape for pure CBAs, MFAs, and their mixtures is hence essential to ensure product quality during storage.

In conclusion, this study emphasizes the significant correlation between the composition of TAGs and the crystallization behavior of different MFAs that were formulated for vegan milk chocolate formulations. The solid-state landscape that can be achieved by blending different TAG mixtures is worth exploring together with the resulting thermal and mechanical properties. This knowledge is essential to overcoming the challenge of finding suitable substitutes for animal fats in confectionary products.

## Supporting Information Available

A Supporting Information file is available free of charge and contains: The Supporting Information is available free of charge at https://pubs.acs.org/doi/10.1021/acs.cgd.5c00227.

Milk fat TAG composition; SAXS patterns during dynamic crystallization experiments; SAXS diffraction peaks as a function of h Miller index for all polymorphs detected; and DSC thermograms collected for pure samples and their mixtures (PDF)
