# Supplementary material for: Crystallization Behavior of Plant-Based Fat Blends Formulated as an Alternative for Anhydrous Milk Fat in Milk Chocolate
Source: Cryst Growth Des. 2025 Apr 4;25(8):2700–16. doi: 10.1021/acs.cgd.5c00227 (PMC12006965; doi:10.1021/acs.cgd.5c00227)
Supplement: Supplementary file 1 — cg5c00227_si_001.pdf [file cg5c00227_si_001.pdf]

Crystallization behavior of plant-based fat blends formulated as alternatives for anhydrous milk fat in milk chocolate

Cecilia Fiore[1], Tom Rutherford[2], Francesca Giuffrida[3], Cynthia Marmet[3], Elena Simone[1,4]*

[1] Department of Applied Science and Technology (DISAT), Politecnico di Torino, Torino, Italy

[2] Nestlé Product Technology Centre Confectionery, Haxby Road, York, YO31 8TA, United Kingdom

[3] Nestlé Research, Vers-chez-les-Blanc, Lausanne 26, 1000, Switzerland

[4] School of Food Science and Nutrition, Food Colloids and Bioprocessing Group, University of Leeds, Leeds, United Kingdom

Corresponding author: *Professor Elena Simone, tel. +39 0110904652, email: elena.simone@polito.it

Section 1. MILK FAT COMPOSITION

Table 1. TAGs composition (%) of the Milk Fat sample used in this work

TAG	%
BuCoP	0.9182
BuCM	0.75215
n/i	3.10407
BuCP	1.93788
BuLaP	1.43687
BuLaO	0.72832
BuMP	4.6425
BuMO	6.09653

**BuPL**	2.26119
**CoMP**	1.88262
**BuPP**	5.90207
**BuPO**	8.28974
**BuOO**	4.07697
**CoPP**	3.18133
**BuPS**	6.56409
**BuSO**	3.26863
**PPCy**	1.48236
**CoSP**	3.92331
**PCyO**	1.35126
**PPC**	1.66113
**PCO**	2.01088
**COO**	0.92472
**LaPP**	1.52086
**PLaO**	2.11061
**PML**	1.08894
**MPP**	1.5502
**PMO**	2.59819
**OMO**	1.86977
**OPL**	0.91094
**PPP**	1.39504
**OPP**	3.10886
**POO**	2.77484
**OOO**	1.13893
**PPS**	0.95853
**SPO**	1.78606
**OOS**	0.87739
**SPS**	0.45176
**SSO**	0.5

Section 2. SAXS PATTERNS

In this section we report the SAXS patterns collected during cooling crystallization experiments. Samples previously inserted in capillaries were heated up from 20°C to 70°C at a rate of 0.5°C/min and kept at such temperature for 5-10 min. A first cooling ramp at -0.5°C/min was set and samples were cooled to 5°C. After 30 min of isothermal hold samples were heated up to 70°c at 5°C/min and then cooled down again to 5°C at -5°C/min (after 5-10 min holding at high temperature). For each figure we report the patterns of the first heating profile (a), the first slow cooling profile (b), the holding period (c), the second heating ramp (d) and the final, fast cooling profile (e).

2.1 CB

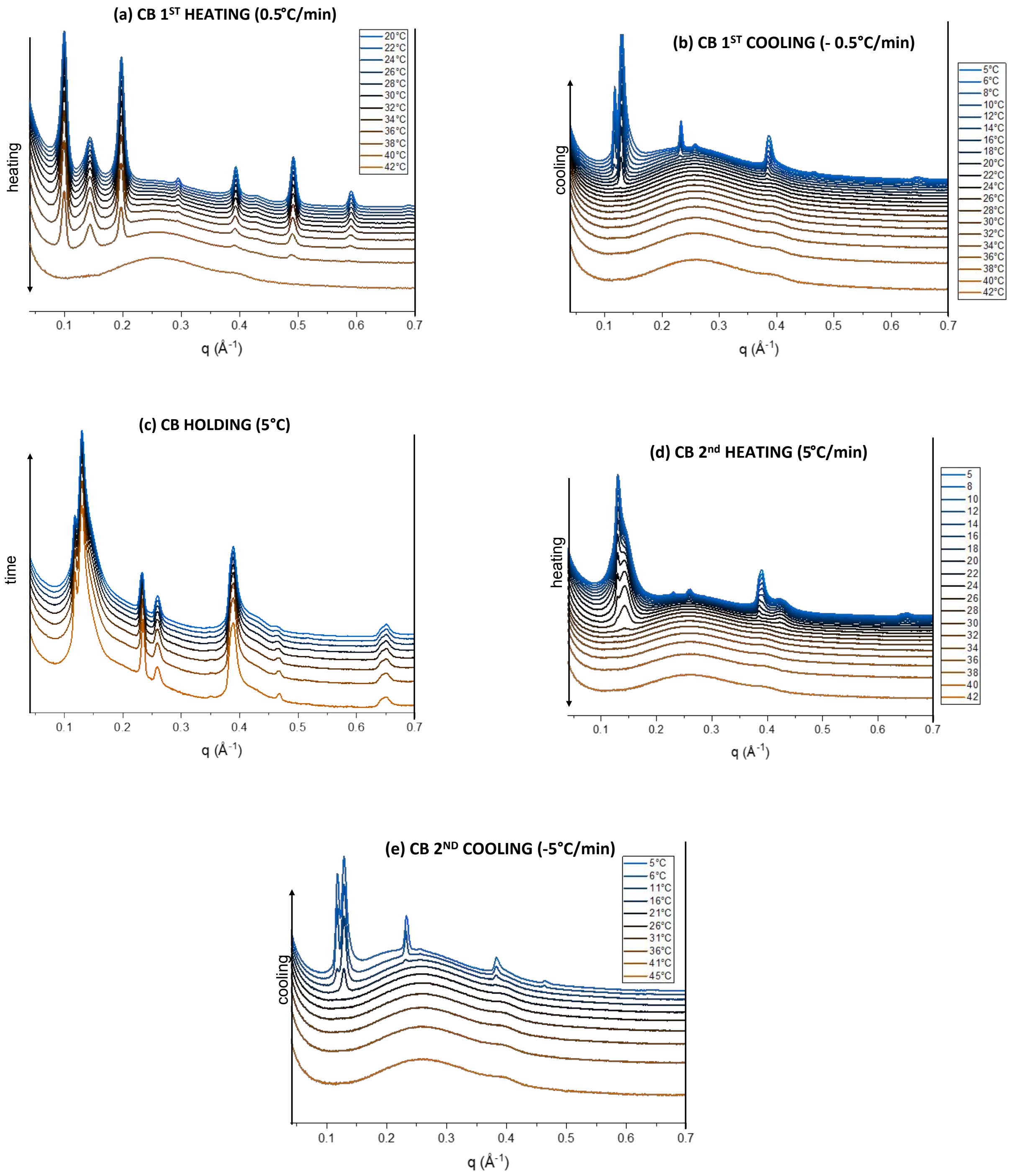

Figure 1. CB SAXS patterns for each temperature profile (a) 1st heating ramp at 0.5 °C/min; (b) 1st cooling ramp at -0.5°C/min; (c) holding at 5°C ; (d) 2nd heating ramp at 5°C/min; (e) 2nd cooling ramp at 5°C/min.

2.2 MF

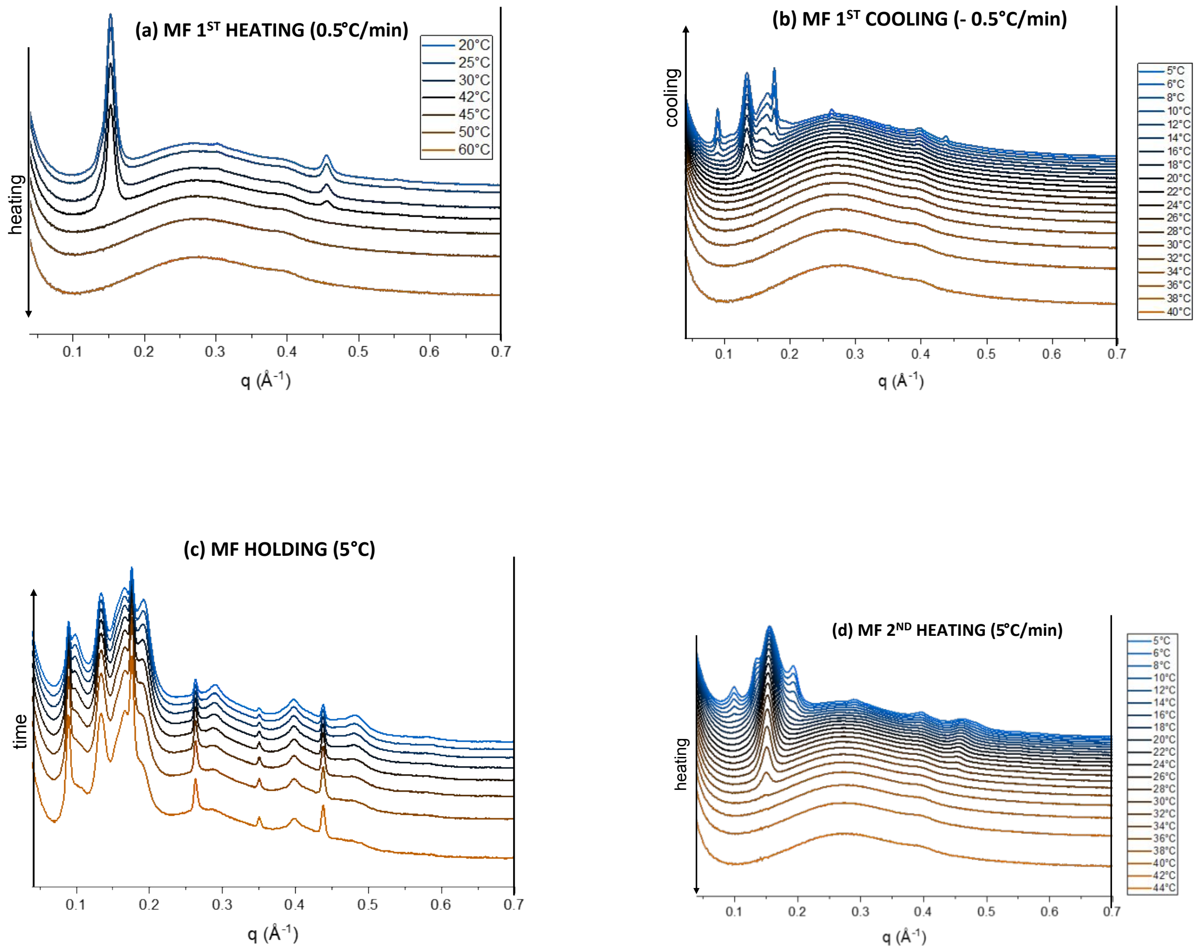

Figure 2 MF SAXS patterns for each temperature profile (a) 1st heating ramp at 0.5 °C/min; (b) 1st cooling ramp at -0.5°C/min; (c) holding at 5°C ; (d) 2nd heating ramp at 5°C/min.

2.3 MF1

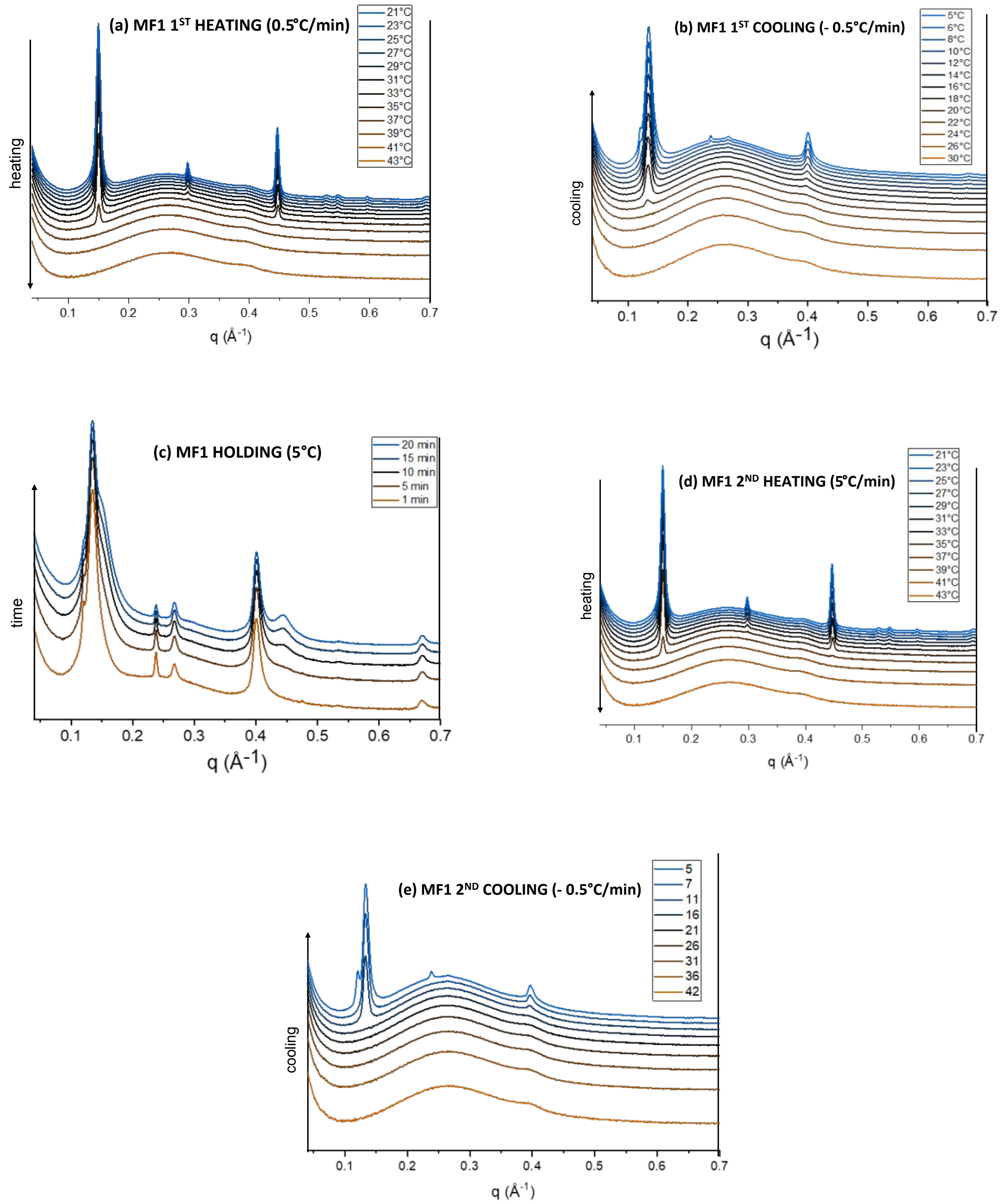

Figure 3. MF1 SAXS patterns for each temperature profile (a) 1st heating ramp at 0.5 °C/min; (b) 1st cooling ramp at -0.5°C/min; (c) holding at 5°C ; (d) 2nd heating ramp at 5°C/min; (e) 2nd cooling ramp at 5°C/min.

2.4 MF2

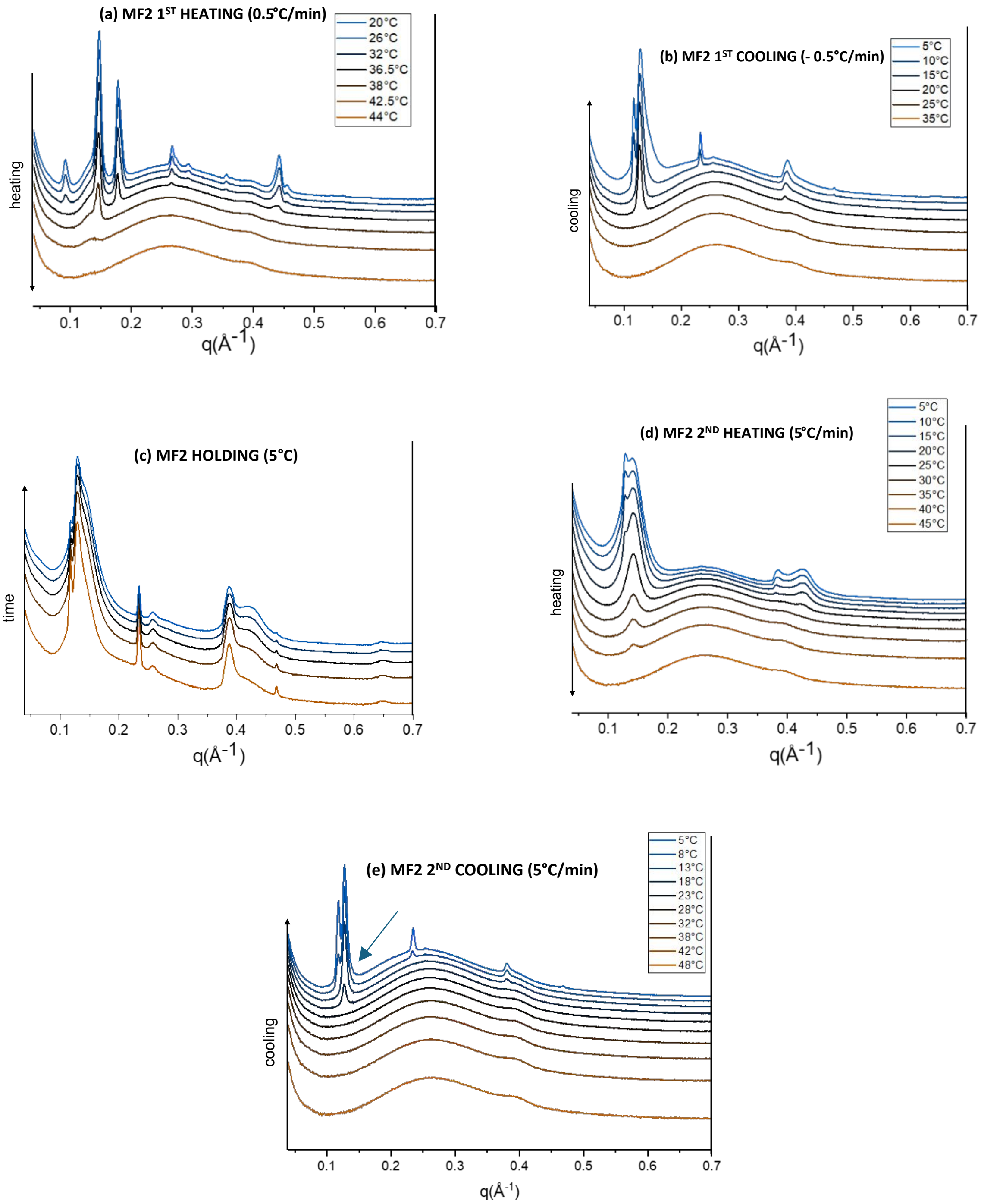

Figure 4 MF2 SAXS patterns for each temperature profile (a) 1st heating ramp at 0.5 °C/min; (b) 1st cooling ramp at -0.5°C/min; (c) holding at 5°C ; (d) 2nd heating ramp at 5°C/min; (e) 2nd cooling ramp at 5°C/min. The blue arrow indicates the second order peak of the $\gamma(3L)$ polymorph, which melted before 20°C.

2.5 MF3

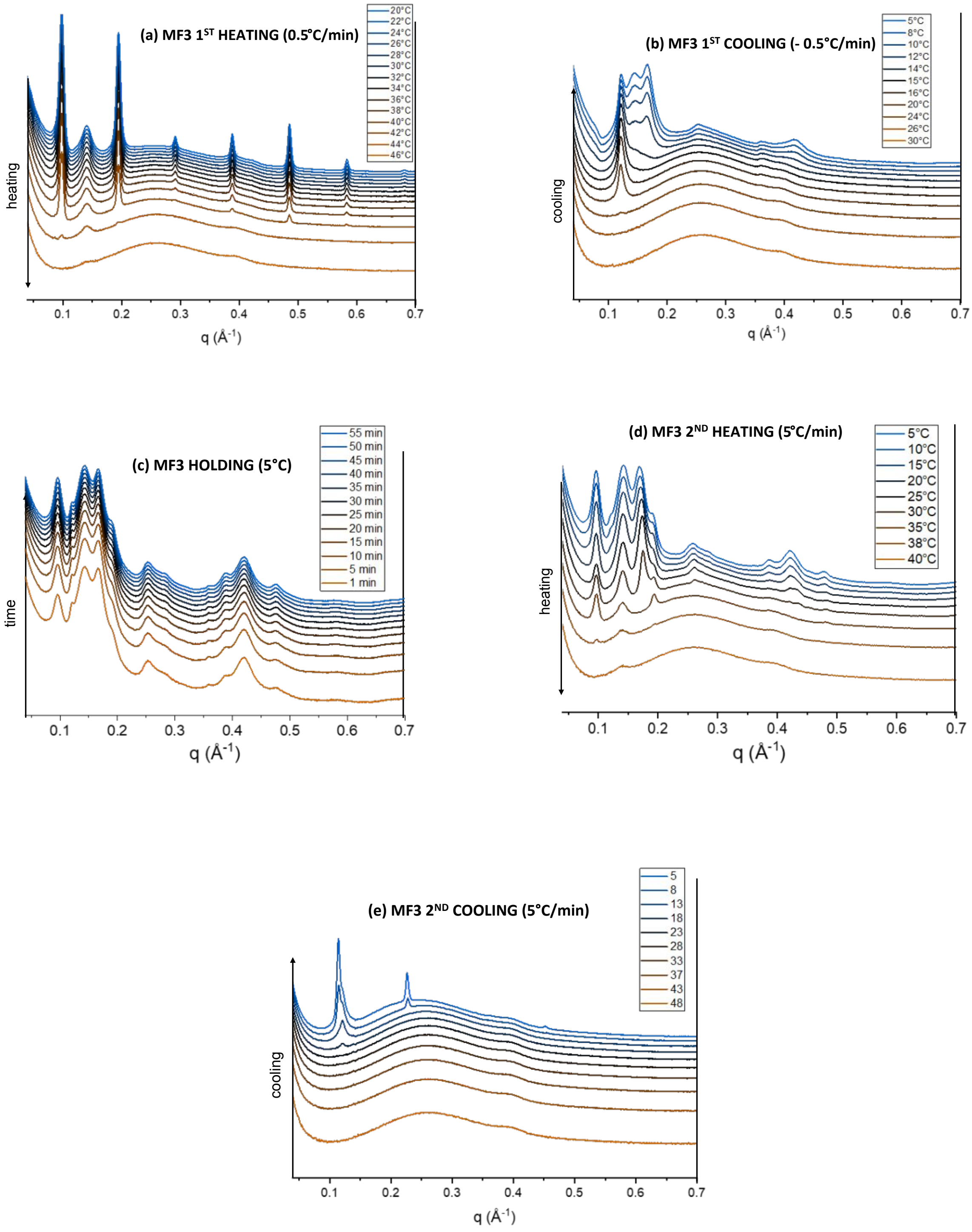

Figure 5 MF3 SAXS patterns for each temperature profile (a) 1st heating ramp at 0.5 °C/min; (b) 1st cooling ramp at -0.5°C/min; (c) holding at 5°C ; (d) 2nd heating ramp at 5°C/min; (e) 2nd cooling ramp at 5°C/min.

2.6 MF4

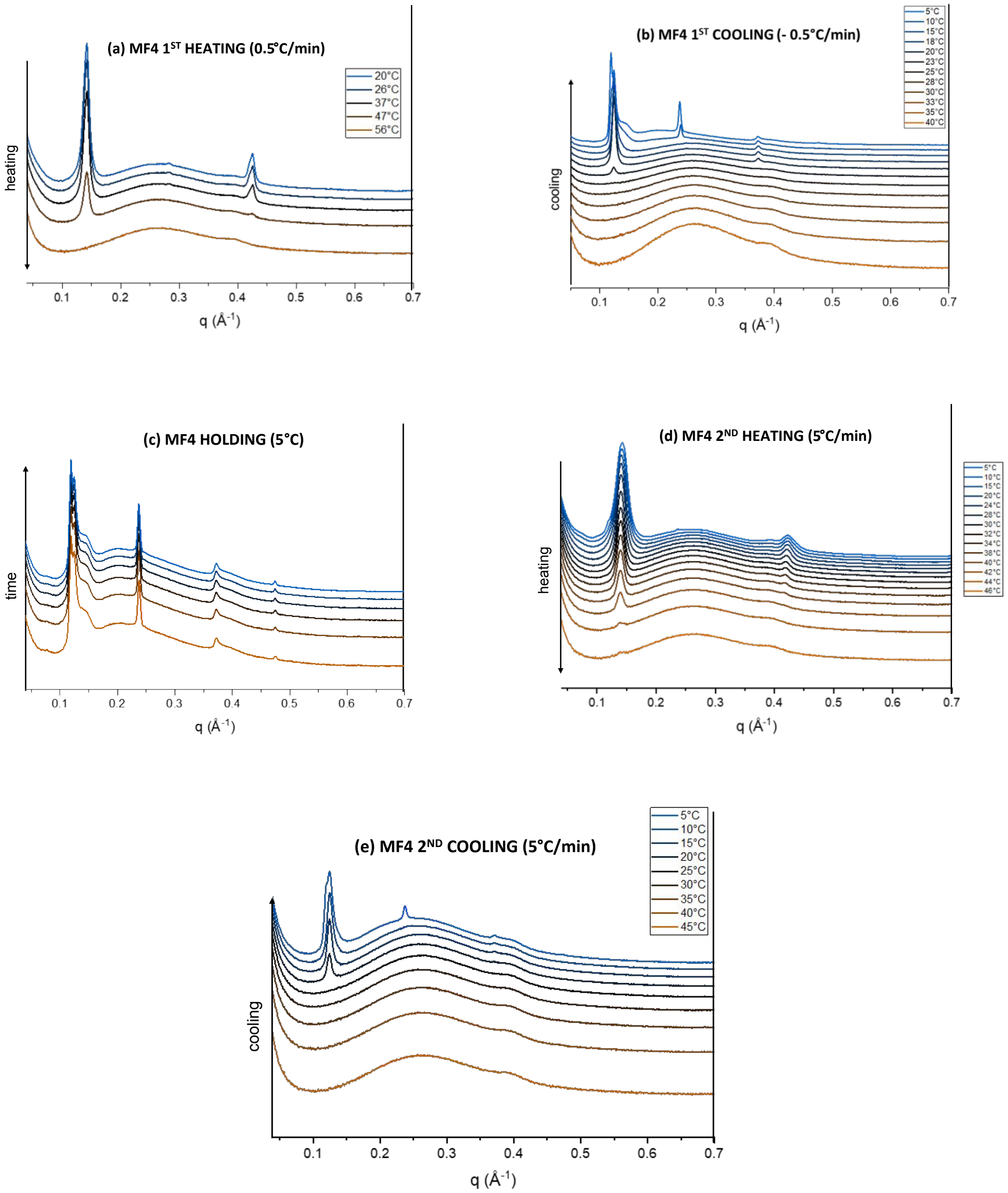

Figure 6. MF4 SAXS patterns for each temperature profile (a) 1st heating ramp at 0.5 °C/min; (b) 1st cooling ramp at -0.5°C/min; (c) holding at 5°C ; (d) 2nd heating ramp at 5°C/min; (e) 2nd cooling ramp at 5°C/min.

2.7 MF 20%

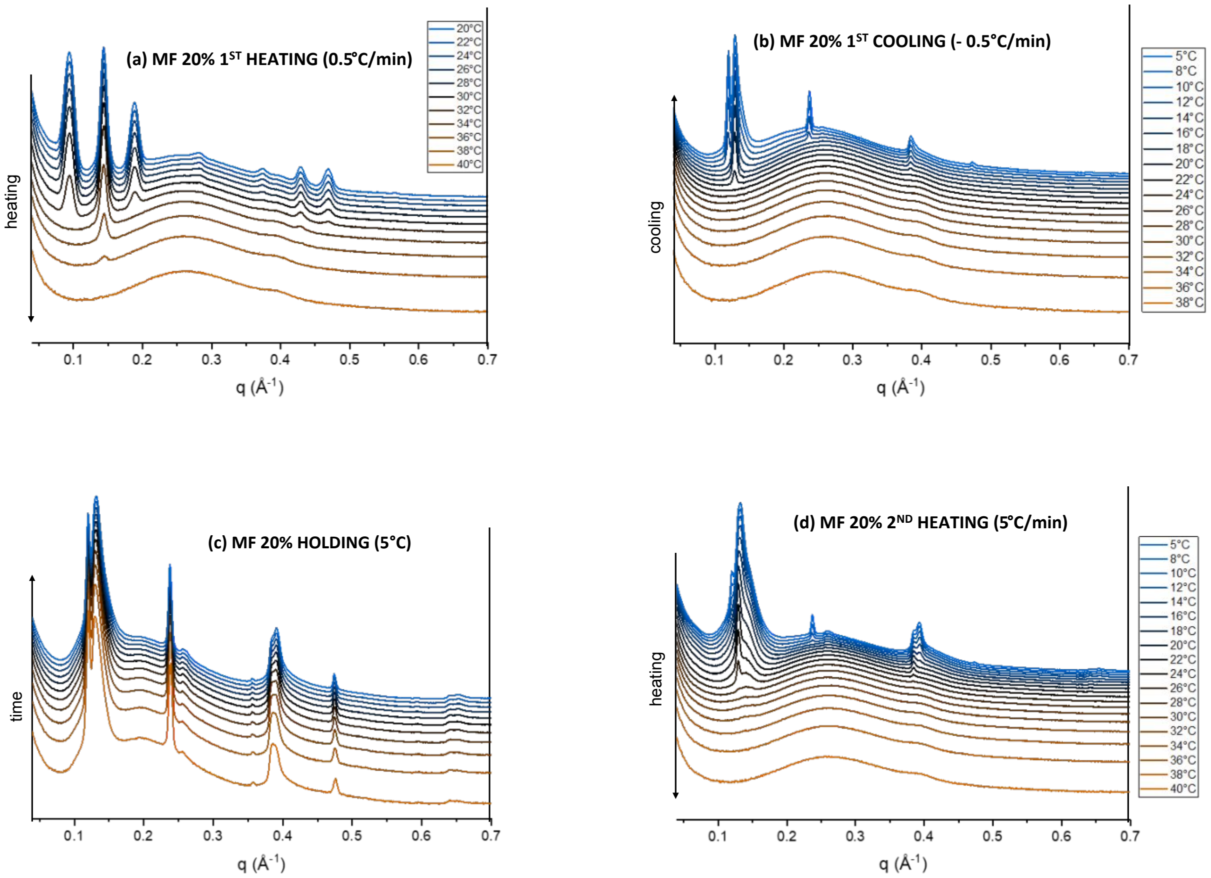

Figure 7. MF 20% SAXS patterns for each temperature profile (a) 1st heating ramp at 0.5 °C/min; (b) 1st cooling ramp at -0.5°C/min; (c) holding at 5°C ; (d) 2nd heating ramp at 5°C/min.

2.8 MF1 20%

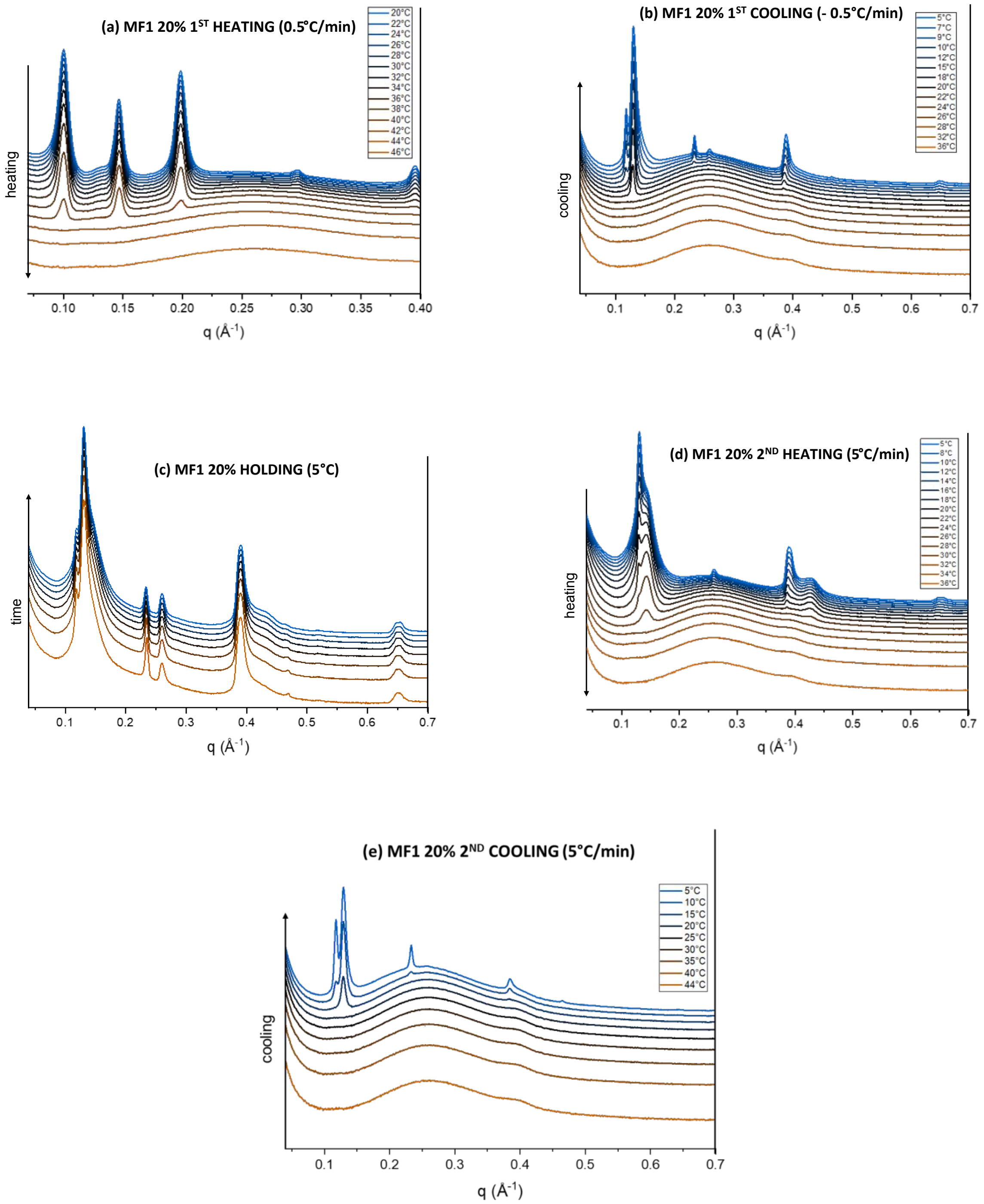

Figure 8. MF1 20% SAXS patterns for each temperature profile (a) 1st heating ramp at 0.5 °C/min; (b) 1st cooling ramp at -0.5°C/min; (c) holding at 5°C ; (d) 2nd heating ramp at 5°C/min; (e) 2nd cooling ramp at 5°C/min.

2.9 MF2 20%

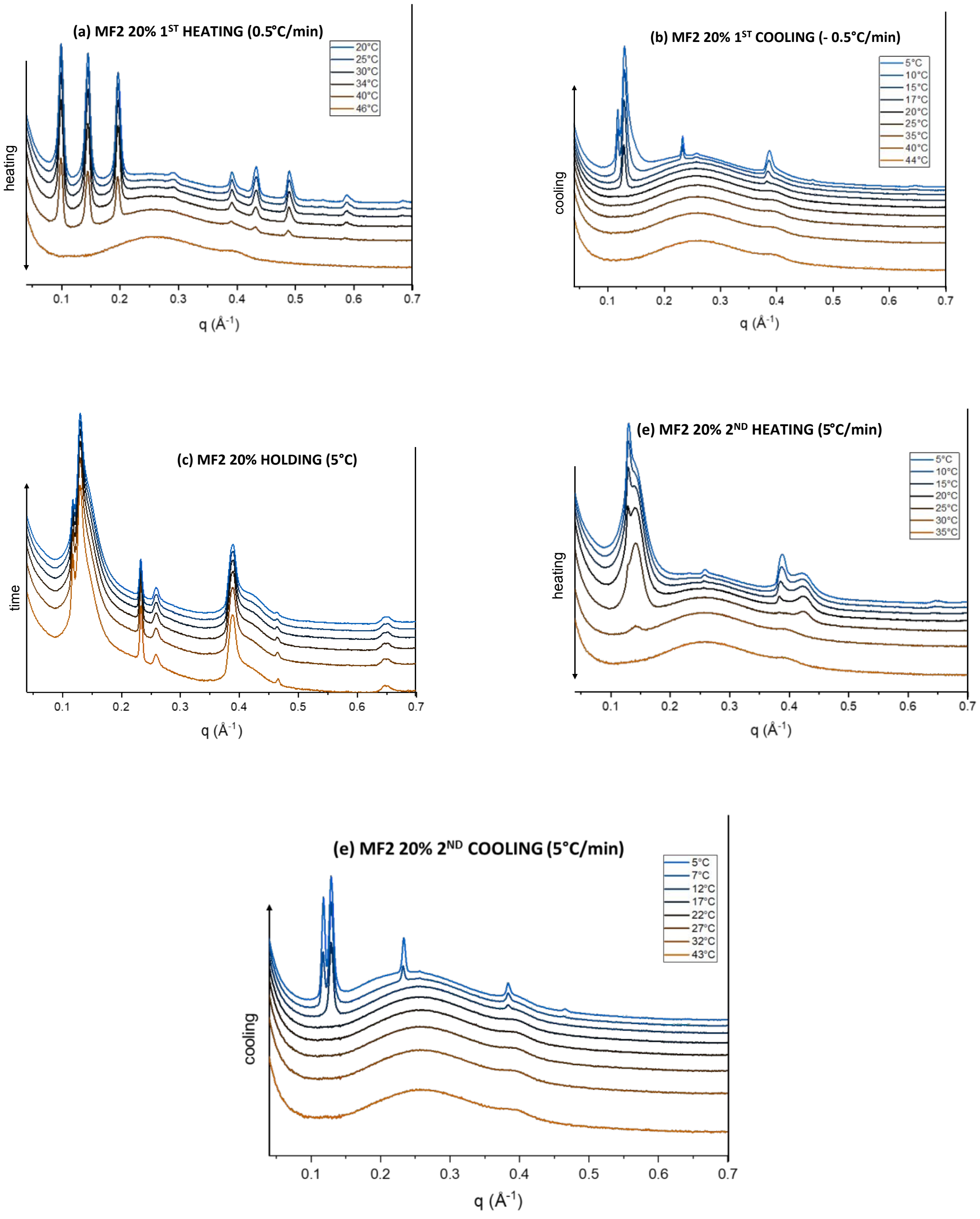

Figure 9. MF2 20% SAXS patterns for each temperature profile (a) 1st heating ramp at 0.5 °C/min; (b) 1st cooling ramp at -0.5°C/min; (c) holding at 5°C ; (d) 2nd heating ramp at 5°C/min; (e) 2nd cooling ramp at 5°C/min.

2.10 MF3 20%

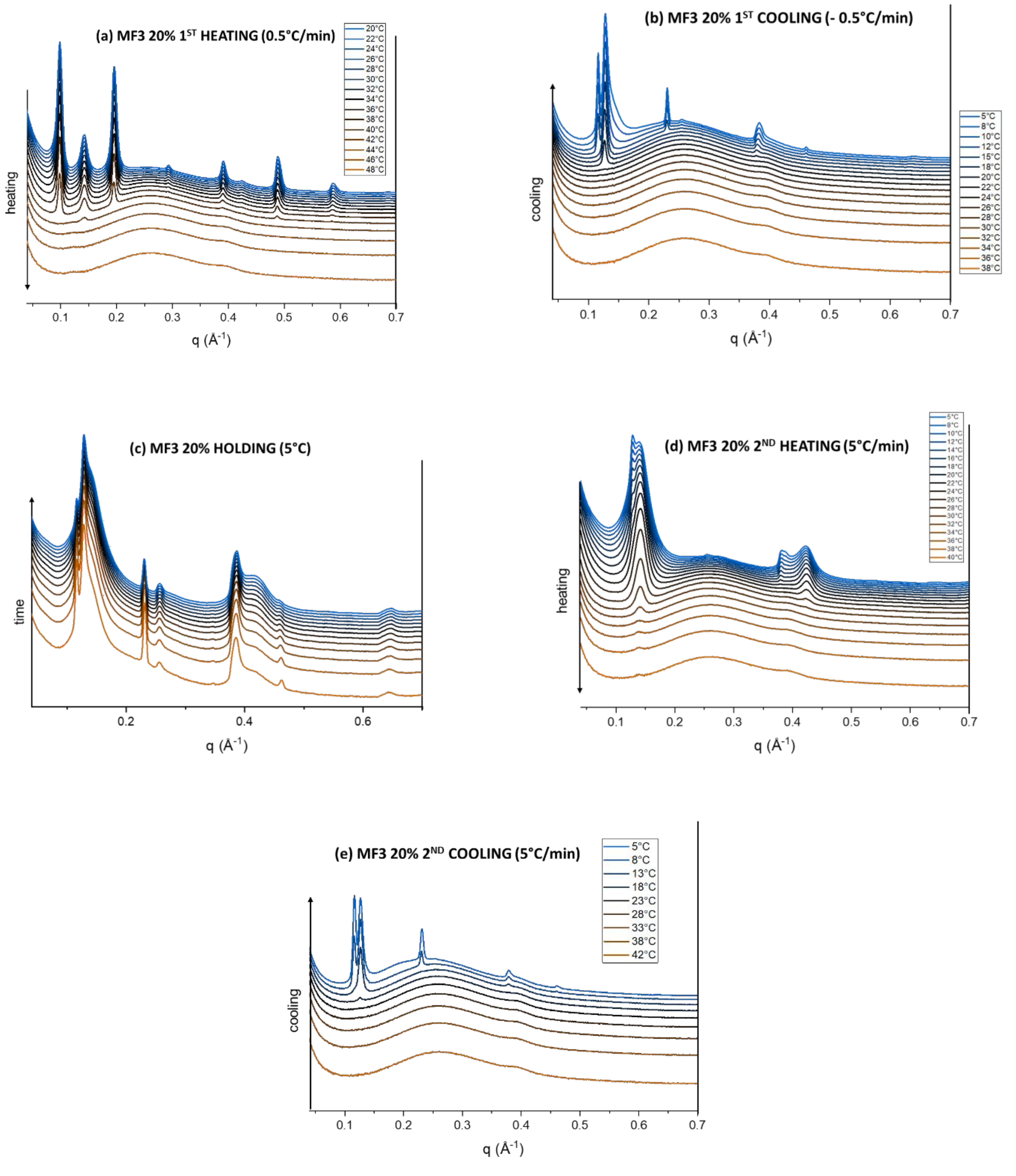

Figure 10 MF3 20% SAXS patterns for each temperature profile (a) 1st heating ramp at 0.5 °C/min; (b) 1st cooling ramp at -0.5°C/min; (c) holding at 5°C ; (d) 2nd heating ramp at 5°C/min; (e) 2nd cooling ramp at 5°C/min.

2.11 MF4 20%

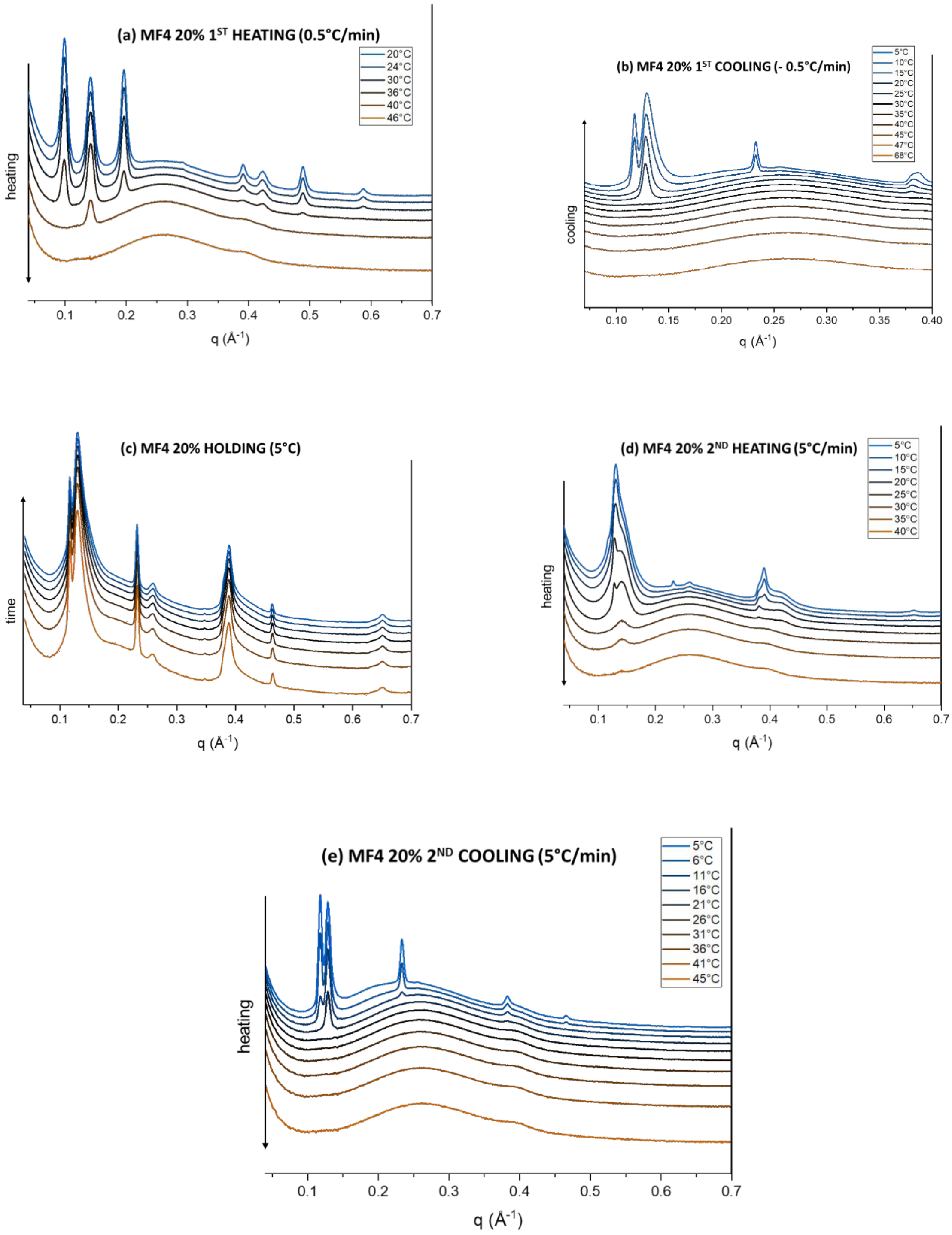

Figure 11. MF4 20% SAXS patterns for each temperature profile (a) 1st heating ramp at 0.5 °C/min; (b) 1st cooling ramp at -0.5°C/min; (c) holding at 5°C ; (d) 2nd heating ramp at 5°C/min; (e) 2nd cooling ramp at 5°C/min.

Section 3. SAXS DIFFRACTION PEAKS AS FUNCTION OF h MILLER INDECES

This sections reports for each sample and section of the thermal profile the q-values recorded in the SAXS patterns. The q-values belonging to the same polymorph are plotted against the corresponding Miller index h value (e.g., consecutive parallel planes). The slope of each line correspond to the d-spacing of the lamellar phase plotted.

3.1 CB

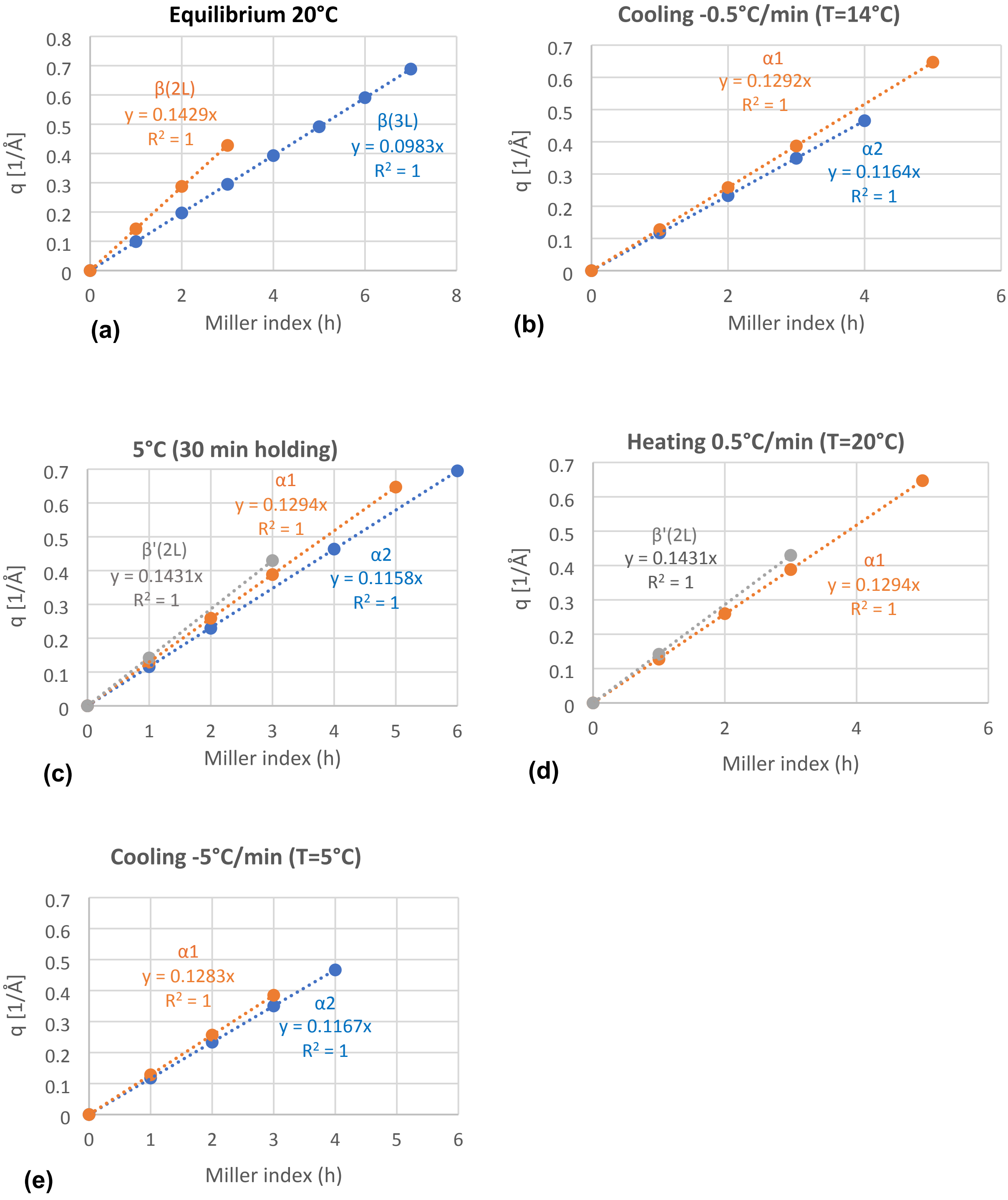

Figure 1. CB-Miller index (h) for SAXS peaks of different diffraction orders during temperature profiles.

3.2 MF

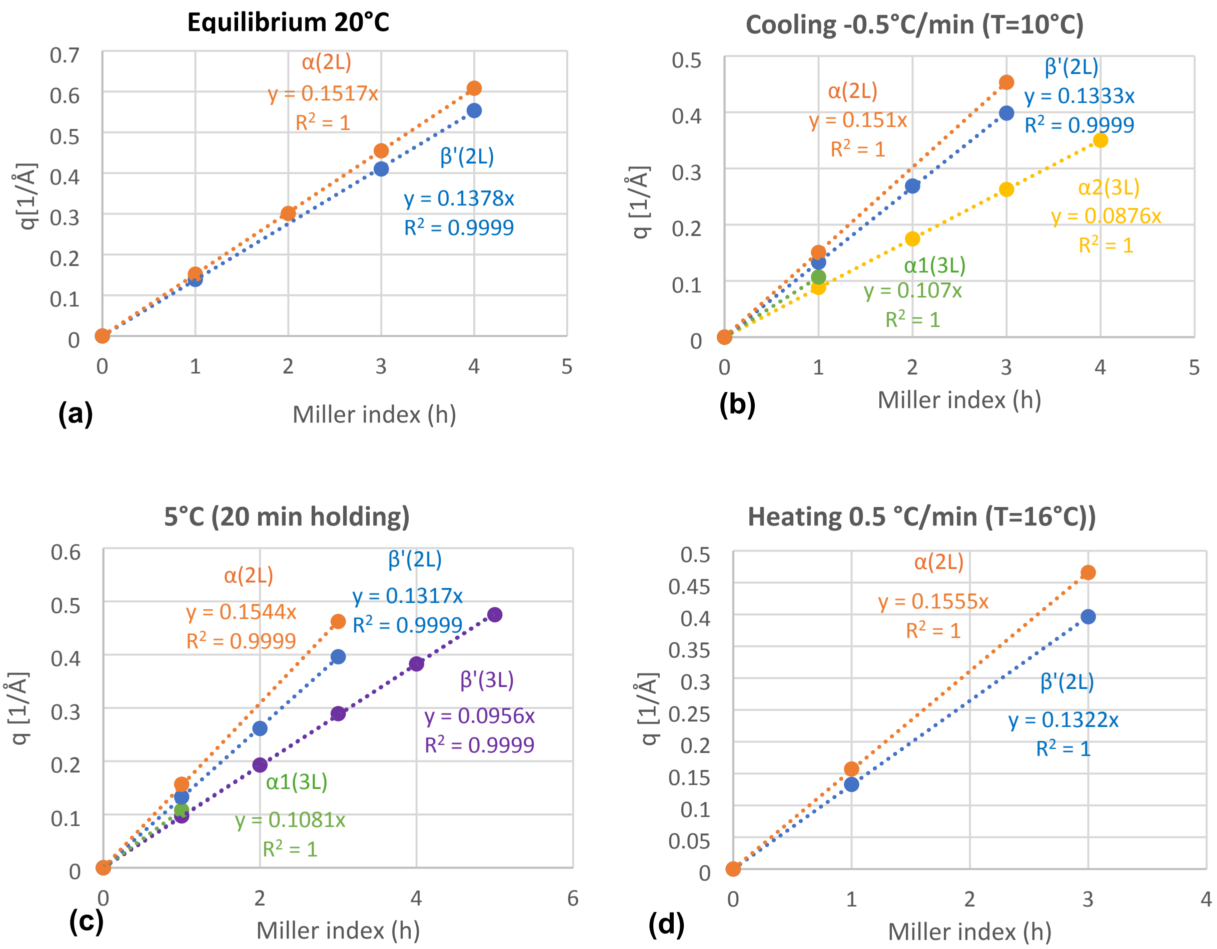

Figure 2. MF- Miller index (h) for SAXS peaks of different diffraction orders during temperature profiles.

3.3 MF1

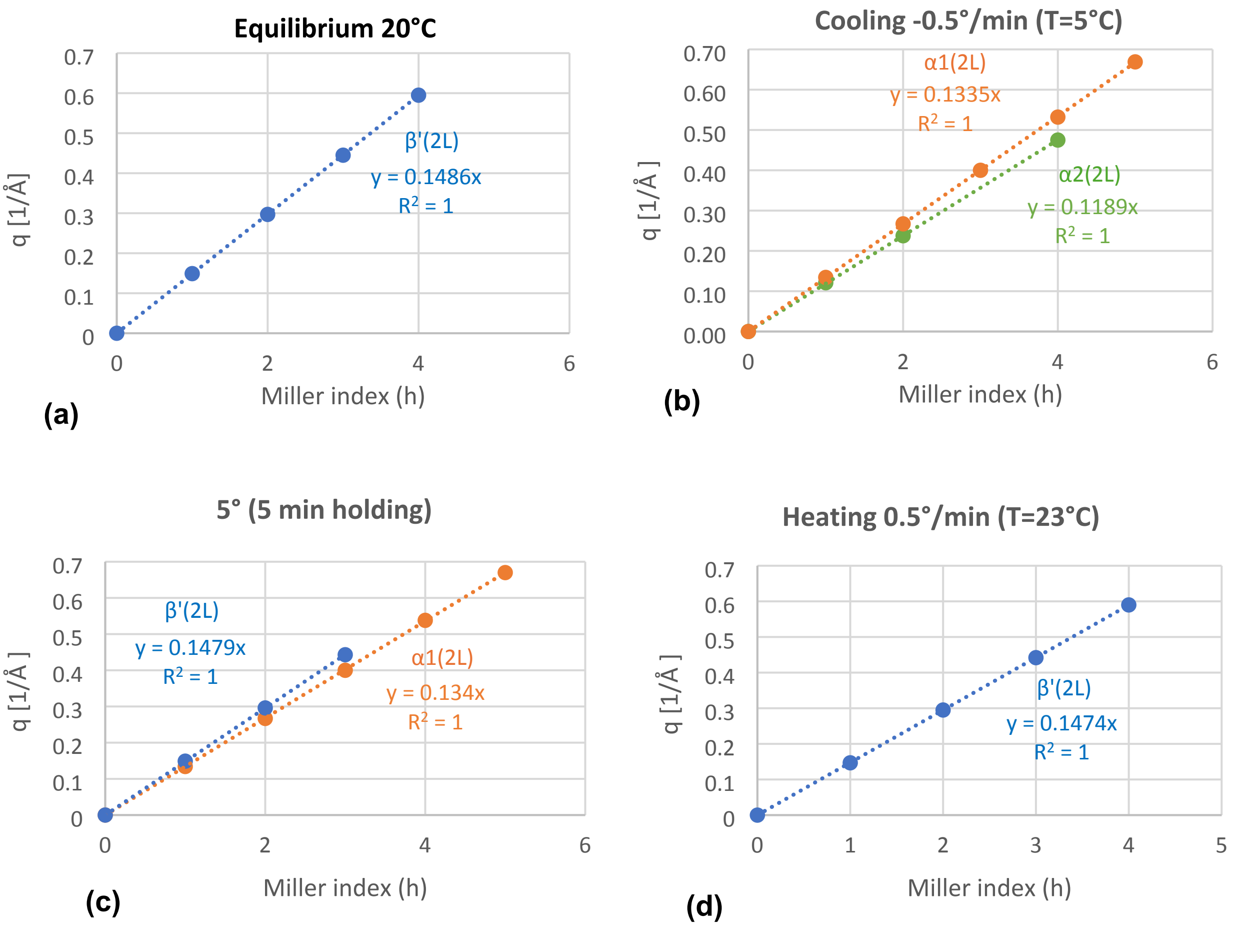

Figure 3. MF1- Miller index (h) for SAXS peaks of different diffraction orders during temperature profiles.

3.4 MF2

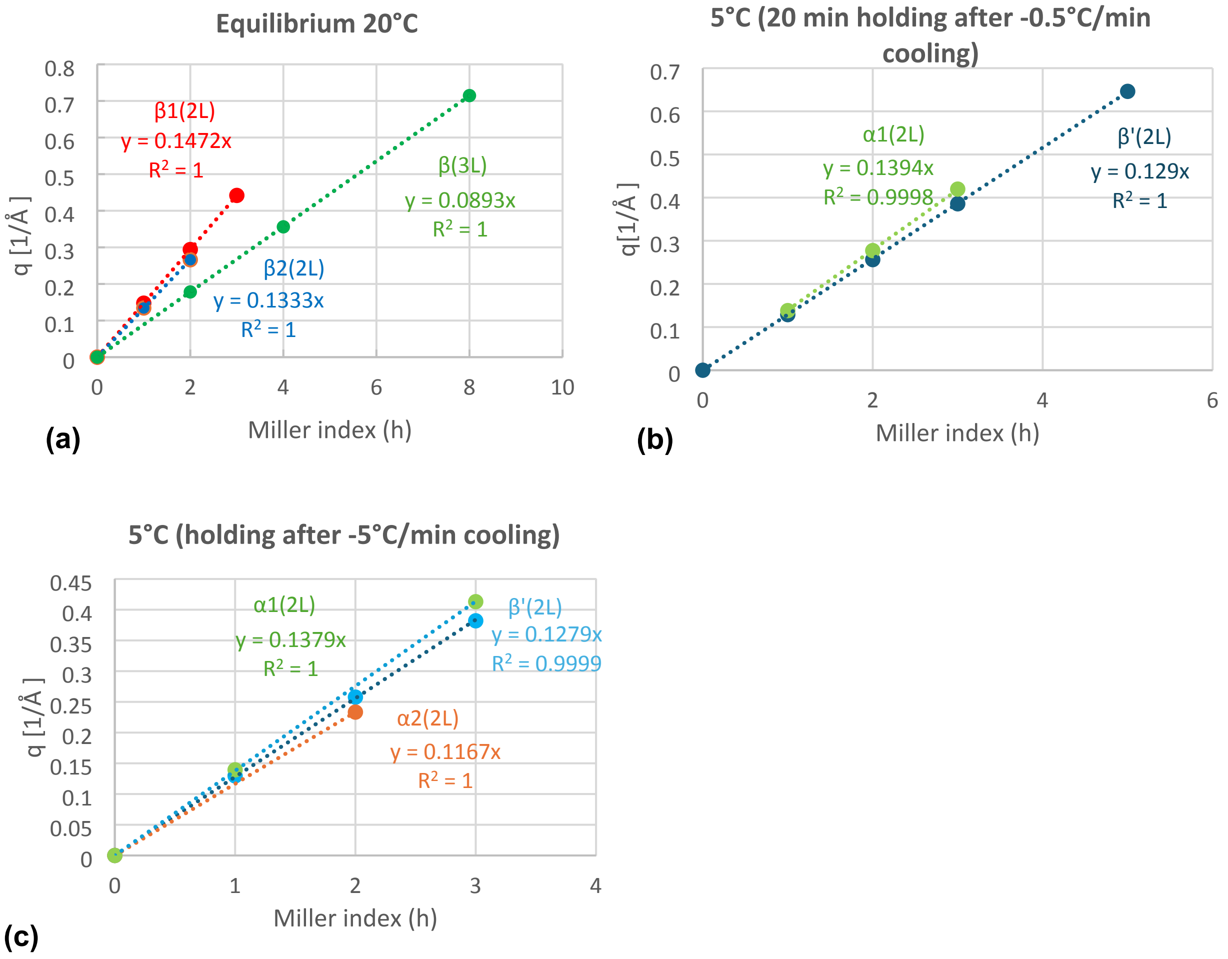

Figure 4. MF2- Miller index (h) for SAXS peaks of different diffraction orders during temperature profiles.

3.5 MF3

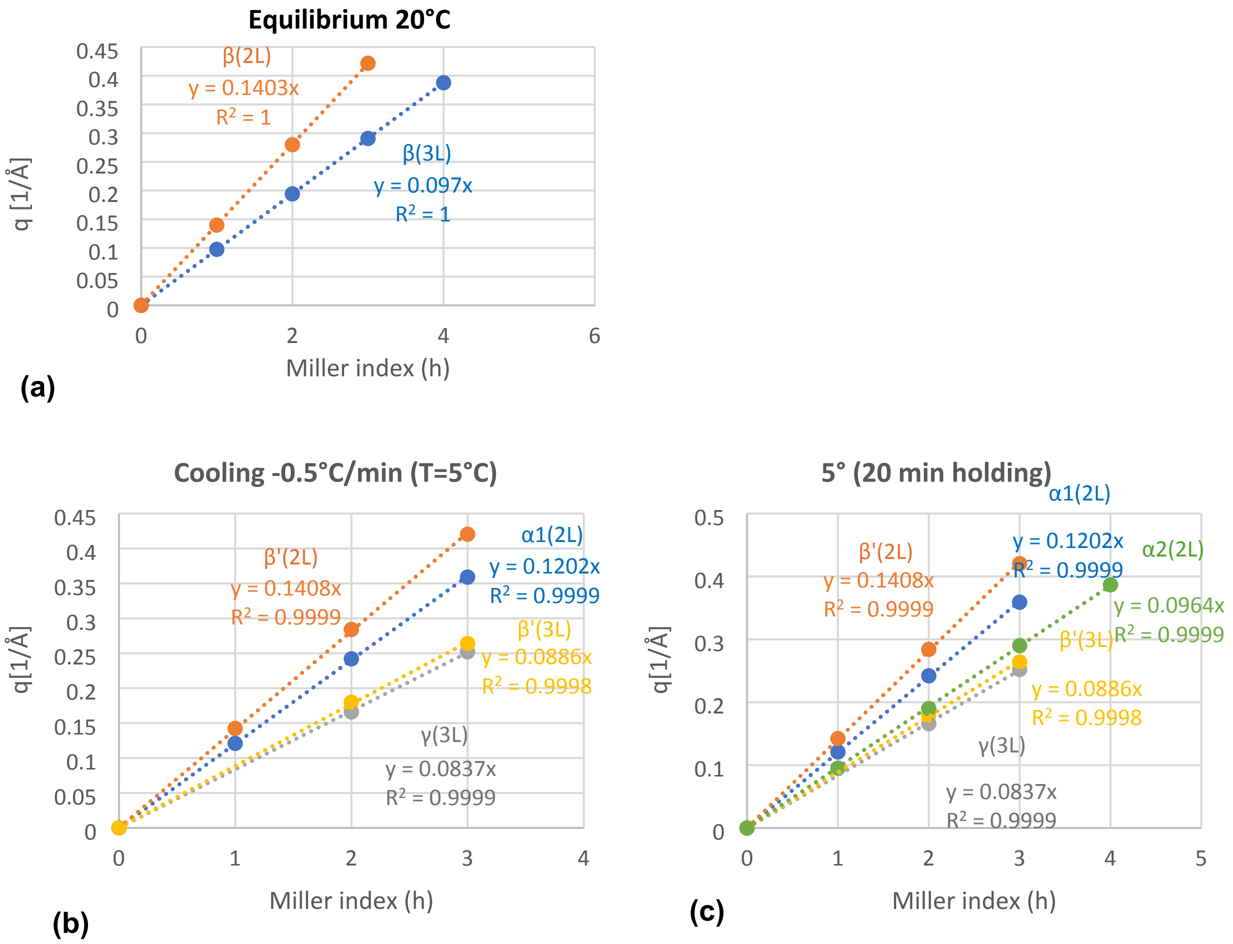

Figure 5 MF3- Miller index (h) for SAXS peaks of different diffraction orders during temperature profiles.

3.6 MF4

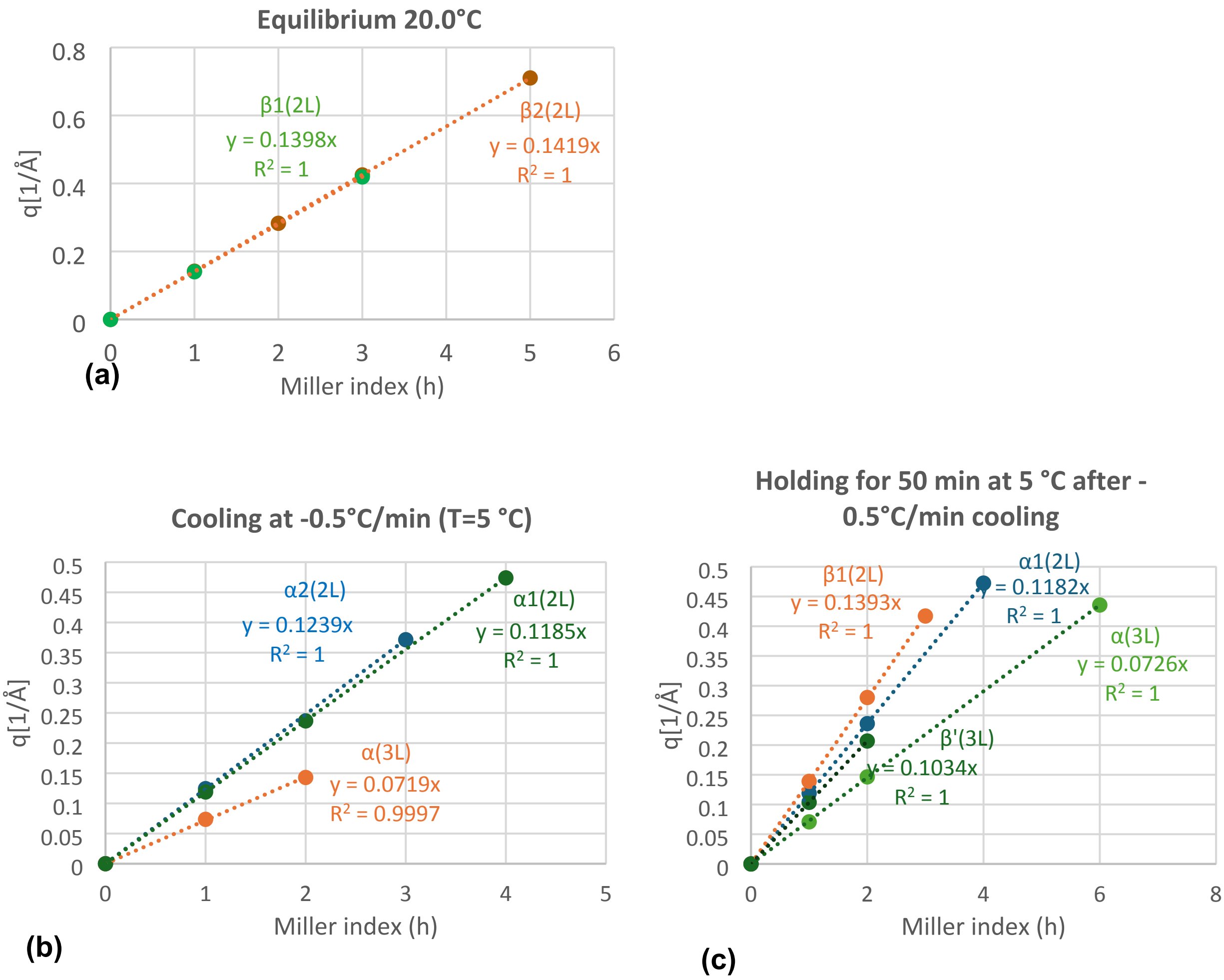

Figure 6 MF4- Miller index (h) for SAXS peaks of different diffraction orders during temperature profiles.

Section 4. DSC thermograms

This section reports the thermograms obtained via DSC analysis for the different TAGs mixtures analysed. Positive peaks indicate endothermic events (endo up). The different colors identify the three steps of the thermal profile: Blue (first cooling ramp at -5°C/min), orange (heating ramp 2°C/min), yellow (second cooling ramp at -2°C/min).

4.1 Cocoa Butter (CB)

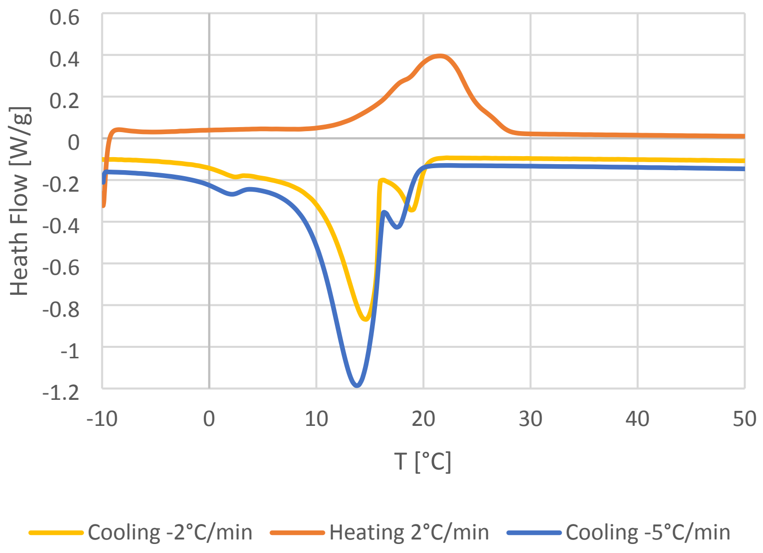

Figure 1. DSC curves of CB during cooling at -5°C/min, heating up at 2°C/min and further cooling at -2°C/min.

4.2 Milk Fat (MF)

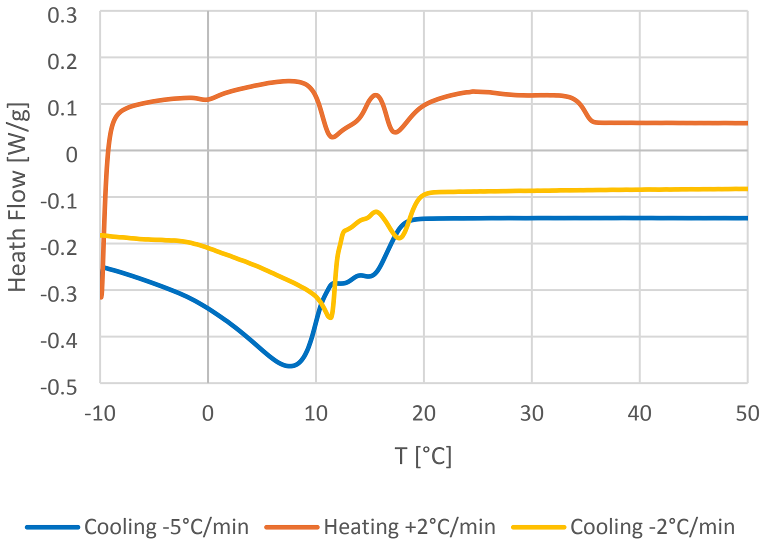

Figure 2. DSC curves of MF during cooling at -5°C/min, heating up at 2°C/min and further cooling at -2°C/min.

4.3 MF1

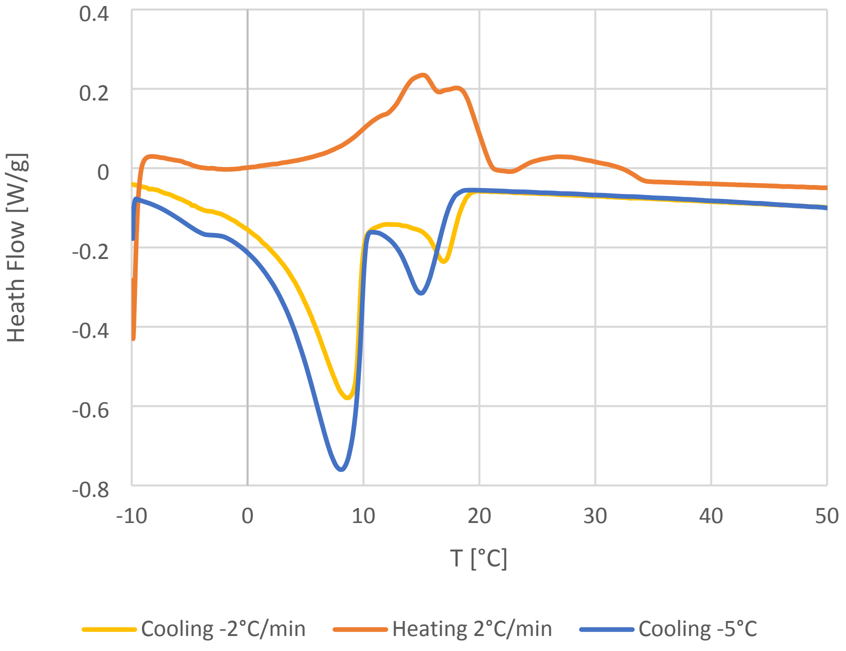

Figure 3. DSC curves of MF1 during cooling at -5°C/min, heating up at 2°C/min and further cooling at -2°C/min.

4.4 MF2

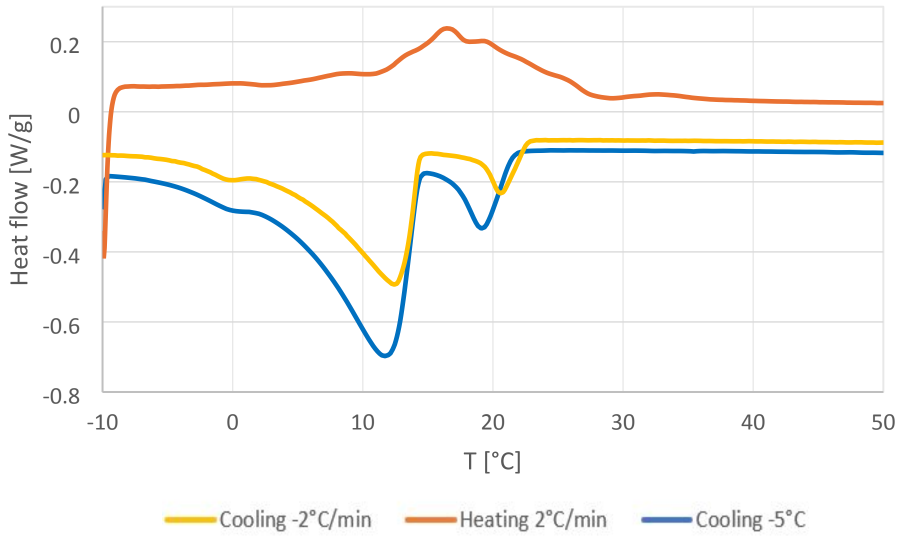

Figure 4. DSC curves of MF2 during cooling at -5°C/min, heating up at 2°C/min and further cooling at -2°C/min.

4.5 MF3

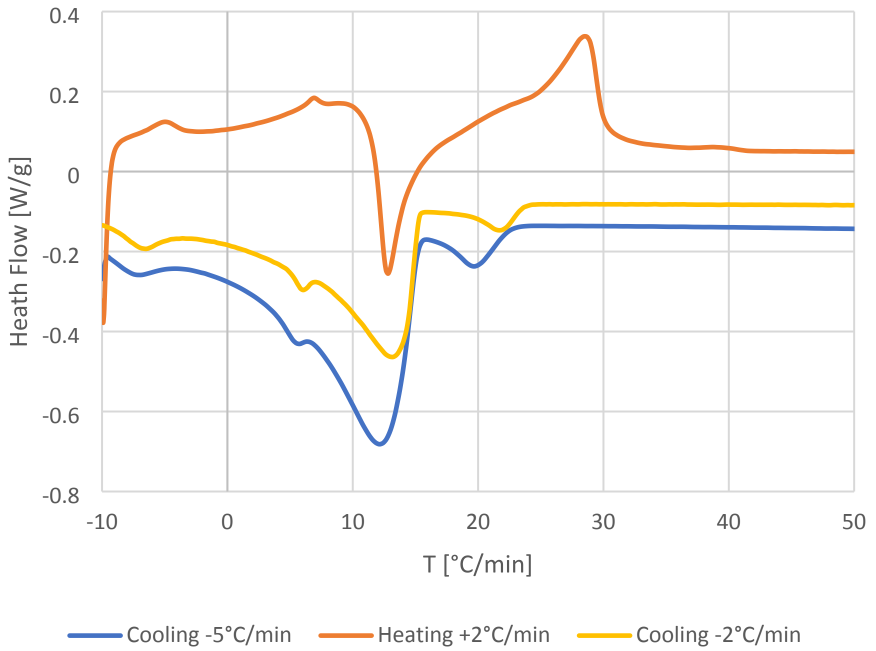

Figure 5. DSC curves of MF3 during cooling at -5°C/min, heating up at 2°C/min and further cooling at -2°C/min.

4.6 MF4

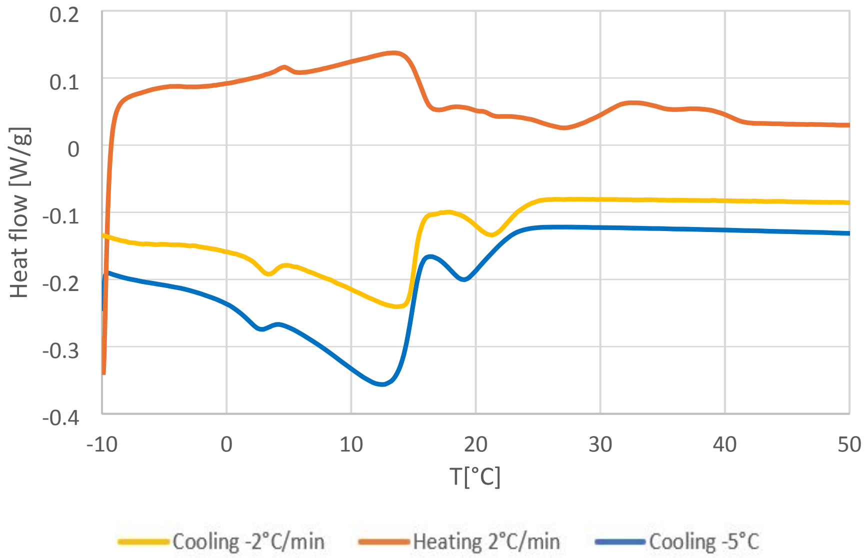

Figure 6. DSC curves of MF4 during cooling at -5°C/min, heating up at 2°C/min and further cooling at -2°C/min.

4.7 MF 20%

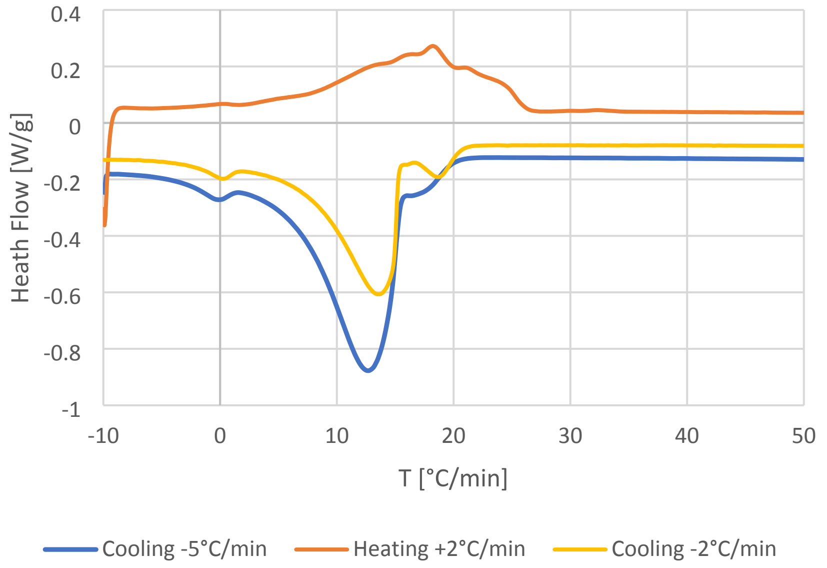

Figure 7. DSC curves of MF 20% during cooling at -5°C/min, heating up at 2°C/min and further cooling at -2°C/min.

4.8 MF1 20%

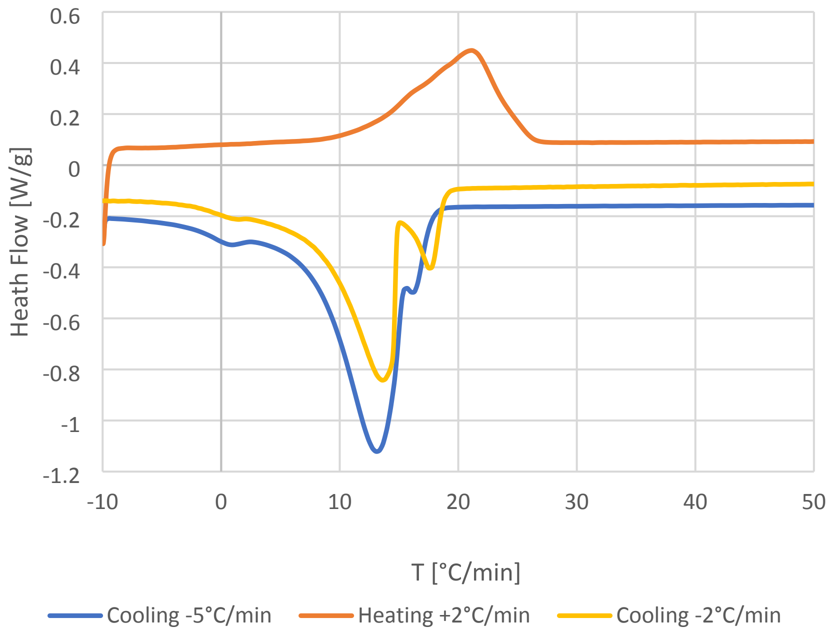

Figure 8. DSC curves of MF1 20% during cooling at -5°C/min, heating up at 2°C/min and further cooling at -2°C/min.

4.9 MF2 20%

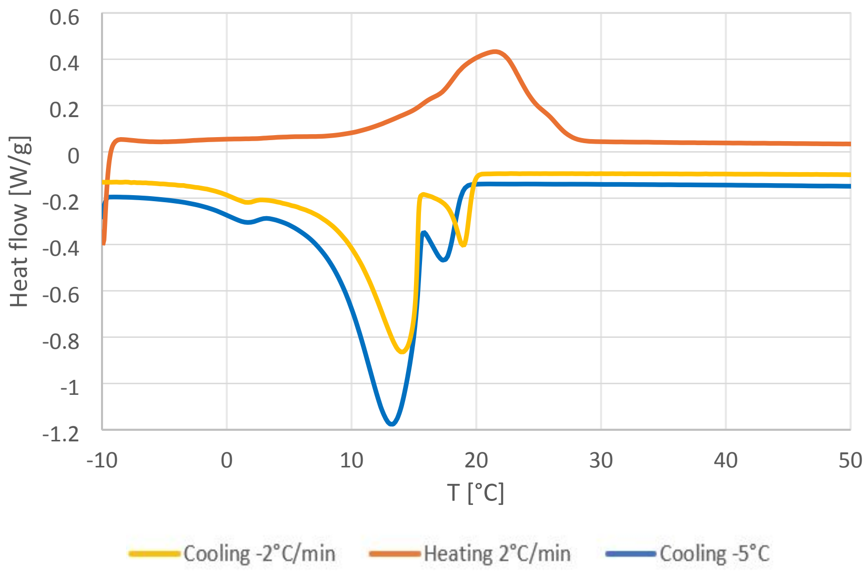

Figure 9. DSC curves of MF2 20% during cooling at -5°C/min, heating up at 2°C/min and further cooling at -2°C/min.

4.10 MF3 20%

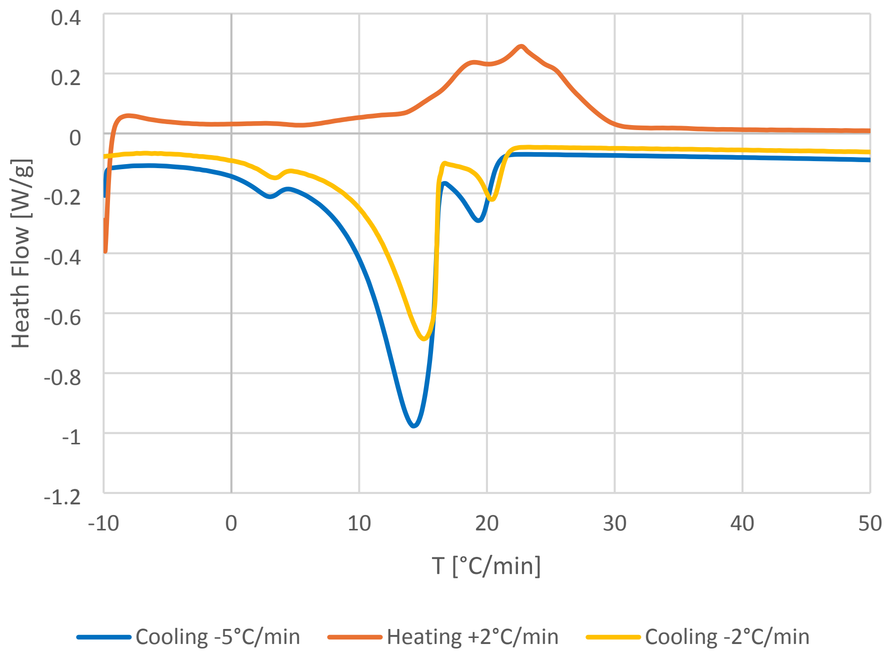

Figure 10. DSC curves of MF3 20% during cooling at -5°C/min, heating up at 2°C/min and further cooling at -2°C/min.

4.11 MF4 20%

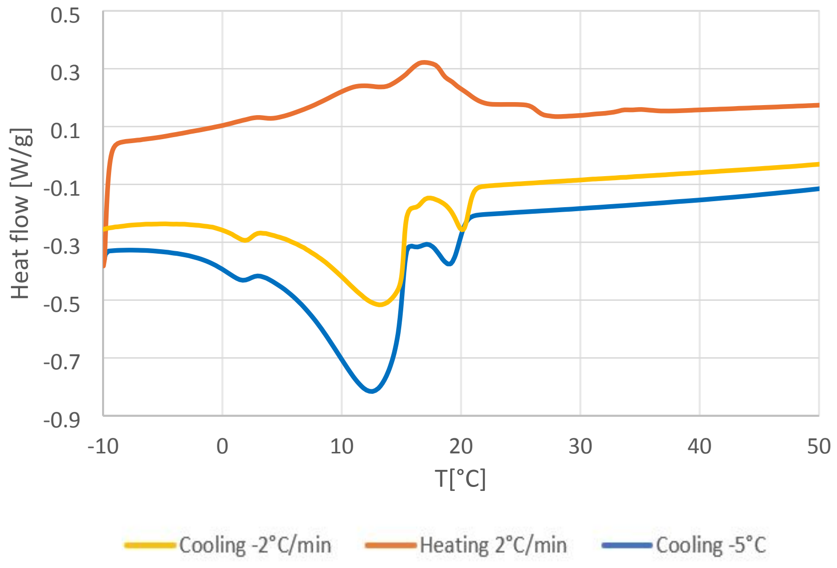

Figure 11. DSC curves of MF4 20% during cooling at -5°C/min, heating up at 2°C/min and further cooling at -2°C/min.
